# Melatonin integrates multidimensional regulation of Na^+^/K^+^-ATPase in ionocytes and promotes stress and ease response in hypoxia-induced air-breathing fish: lessons from integrative approach

**DOI:** 10.3389/fphys.2022.1012729

**Published:** 2023-01-12

**Authors:** M. C. Subhash Peter, R. Gayathry, S. Simi, Valsa S. Peter

**Affiliations:** ^1^ Inter-University Centre for Evolutionary and Integrative Biology-ICEIB, School of Life Sciences, University of Kerala, Kariavattom, Thiruvananthapuram, India; ^2^ Department of Zoology, University of Kerala, Kariavattom, Thiruvananthapuram, India

**Keywords:** melatonin, fish, stress response, ease response, Na+/K+ ATPase, hypoxia, ionocyte, ionosomotic regulation

## Abstract

As circadian regulator, melatonin is involved in many physiological processes including ionosmotic regulation in fishes. Na^+^/K^+^-ATPase (NKA), an ubiquitous Na^+^/K^+^ transporter in ionocyte epithelia that drives electrochemical Na^+^ gradients and systemic osmotic integration, is a target of stress in fish. However, it is not certain how melatonin regulates NKA functions in ionocyte epithelia and how it modulates the adaptive response such as stress and ease response in fish particularly in hypoxia condition. We, thus, examined the short-term *in vivo* action of melatonin on the dynamics of NKA regulation in branchial, renal and intestinal ionocytes of hypoxia-induced air-breathing fish (*Anabas testudineus* Bloch). Interestingly, we found a rise in plasma melatonin in fish when kept for 30 min of forced submergence in water and that indicates a role for melatonin in hypoxia tolerance. A fall in blood [Na^+^_,_ K^+^] occurred in these hypoxic fish which later showed a recovery after melatonin treatment. Similarly, melatonin favored the fall in NKA activity in branchial and renal epithelia of hypoxic fish, though it remarkably stimulated its activities in non-stressed fish. Likewise, melatonin that produced differential pattern of mRNA expression in nkaα1-subunit isoforms (*nkaα1a, nkaα1b and nkaα1c*) and melatonin receptor isoforms (*mtnr1a, mtnr1bb, mtnr1bb*_*x1x2*_) in the tested ionocyte epithelia, showed reversed expression in hypoxic fish. In addition, the rise in NKAα-protein abundance in branchial and renal epithelia of melatonin-treated hypoxic fish indicated a recovery action of melatonin. A higher NKAα-immunoreactivity was found in the immunohistochemical and immunofluorescent images of branchial ionocytes and renal proximal and distal ionocytes of hypoxic fish treated with melatonin. Furthermore, an activation of PKA and PKG-dependent phosphorylation was found in branchial epithelia of hypoxic fish. The generated integrative parabola model showed that melatonin has a maximum targeted action on NKA function in the renal epithelia, suggesting its lead role in the integration of ionosmotic balance during the recovery or ease response. Over all, the data indicate a multidimensional and preferential action of melatonin on NKA regulation in fish ionocytes that integrate the recovery action against hypoxia, thus pointing to a major role for melatonin in stress and ease response in this fish.

## 1 Introduction

As a potent regulator of circadian and seasonal rhythms, the pineal neurohormone melatonin is involved in several physiological activities in vertebrates including fishes (Velarde et al., 2010; Esteban et al., 2013; Mukherjee et al., 2014; Mukherjee and Maitra, 2015; Muñoz-Pérez et al., 2016; Sánchez-Vázquez et al., 2019). This multifunctional hormone has a control on the regulation of ionosmotic homeostasis in fishes (Kulczykowska, 2002; Kulczykowska et al., 2006; Falcon et al., 2007; Sangiao-Alvarellos et al., 2007; Ngasainao and Lukram, 2016). For example, melatonin is known to influence water and mineral balance in mammals (Tsuda et al., 1995; Drew et al., 2001; SajeebKhan and Peter, 2015), birds (Ching et al., 1999) and amphibians (Wright et al., 1997). The widespread distribution of melatonin receptors in the osmoregulatory tissues of both freshwater and seawater fishes indicates that these tissues are the possible sites for melatonin action (Kulczykowska, 2002; Kulczykowska et al., 2006). Moreover, studies have reported that salinity can modify the melatonin contents in fish intestine and gills along with its levels in plasma (Lopez-Olmeda et al., 2009), pointing to a role for extra-pineal source in melatonin homeostasis in fish. Melatonin as an evolutionary conserved hormone plays a major role in the adaptive behavior of the animal to the environment and is known to regulate the daily and annual physiological rhythms and growth of zebrafish (Lima-Cabello, 2014). Despite the reports that demonstrate an action of melatonin in fishes (Kulczykowska et al., 2006; Sangiao-Alvarellos et al., 2007; Esteban et al., 2013; Lunkes et al., 2021), an integrative action of melatonin on Na^+^/K^+^ATPase (NKA) regulation in ionocytes especially in hypoxia-induced stress is not yet known particularly in air-breathing fish.

The integration of ion transport activities of ionocytes in the osmoregulatory epithelia of gills, kidney and intestine is critical for maintaining osmointegration and systemic ion homeostasis (Evans et al., 2005; Peter, 2011) in bony fishes. The branchial ionocytes, popularly known as chloride cells or mitochondrial rich cells, are the integral part of branchial epithelia and act as the major site for ion and water exchange in teleosts (Evans et al., 2005) including zebrafish (Guh and Hwang, 2017) and air-breathing fish (Rejitha et al., 2009; Peter et al., 2011). Likewise, renal ionocytes that are located in the proximal, distal and collecting tubules of kidney and the enteric ionocytes in the intestinal epithelia are equally involved in the regulation of systemic Na^+^ homeostasis (Peter et al., 2022). These ionocytes in the osmoregulatory epithelia possess a transmembrane ion transporter NKA that drives electrochemical Na^+^ gradient and membrane potential of cell, contributes to the osmotic regulation of cell volume (Mobasheri et al., 2000) and ion homeostasis (McCormick, 2001; Peter and Gayathry, 2021). In addition, many secondary transport systems that are mainly localized in the apical and basolateral membranes of osmoregulatory epithelia are controlled by NKA (Skou and Esmann, 1992; Tipsmark et al., 2011).

Structurally, NKA consists of three subunits α, β and γ (FXYD protein) in which α and β are necessary for ion pumping and that dimerize to form functional NKA, while the third subunit regulates the pumping function (Kaplan, 2002; Jorgensen et al., 2003; Garty and Karlish, 2006). The catalytic α-subunit is a large protein of 110 kDa, containing binding sites for ions, ATP and ouabain and a phosphoryation site (Skou and Esmann, 1992; Therien and Blostein, 2000; Laursen et al., 2013), and the β-subunit interacts with the α-subunit and is involved in regulating ion-binding affinity of the enzyme complex (Blanco and Mercer, 1998). Four α-subunits (α1, α2, α3 and α4) and three β-subunits (β1, β2 and β3) have been identified in vertebrates with varying tissue distributions (Blanco and Mercer, 1998; Lingrel, 2010). Numerous studies have proved the presence of these isoforms in teleosts; particularly the catalytic α-subunit (Tipsmark et al., 2011; Urbina et al., 2013; Ching et al., 2015; Yang et al., 2016) and the importance of α-subunit in osmoregulation are well studied (Blondaeu-Bidet et al., 2016). In fishes, only three isoforms (α1, α2 and α3) have been identified, among which α1 is of particular importance in osmoregulation due to its high expression in osmoregulatory tissues (Tipsmark et al., 2011; Ip et al., 2012; Urbina et al., 2013; Armesto et al., 2014; Moorman et al., 2015). In addition, Ip and coworkers (2012) reported the presence of *nkaα1* sub isoforms *viz. nkaα1a, nkaα1b* and *nkaα1c* in the gills of climbing perch *Anabas testudineus*. Several studies have provided evidence that the short-term regulation of NKA activity occurs as a direct effect on the kinetic behavior of the enzyme *via* the phosphorylation or dephosphorylation process of the catalytic unit (McDonough and Farley, 1993; Chibalin et al., 1998; 1999).

In fishes, induction of stress evokes a set of stress response that includes osmotic and ion transport disturbances (Wendelaar Bonga, 1997; Peter and Peter, 2011). The air-breathing fish *Anabas testudineus* relays on many ion transporters for maintaining ion and osmotic balance (Peter and Peter, 2011). Extensive studies have shown that when kept at water immersion for 30 min this fish underwent hypoxia with a modified regulatory dynamics of NKA at transcriptomic level as evident in the altered pattern of mRNA expression of *nkaα1* sub isoforms such as *nkaα1a, nkaα1b* and *nkaα1c* in the osmoregulatory tissues (Simi et al., 2017; Peter and Gayathry, 2021). It has been documented that upon stressor exposure, fish develops a cycle of adaptive response that produces both stress and ease response (Peter, 2013; Peter et al., 2022). With the classic disturbance in physiological mechanisms that include a disturbed ion homeostasis, fishes evoke stress response along with the release of stress hormones such as adrenaline and cortisol (Wendelaar Bonga, 1997). Alternatively, fishes have also developed mechanism to ameliorate the magnitude of stress response by evoking recovery or ease response in order to regain the basal homeostatic status with the aid of neuroendocrine/cytogenic signals like melatonin and nitric oxide (Peter, 2013; Peter and Gayathry, 2021; Peter et al., 2022). Similarly, beneficial role of melatonin in lowering the magnitude of stress response has been proposed in many fishes (Sánchez-Vázquez et al., 2019; Yang et al., 2021a; Kruk et al., 2021). In addition, anti-stress and anti-depressant role of melatonin have been reported in higher vertebrates (Konakchieva et al., 1997; Paredes et al., 2007; Detanico et al., 2009; SajeebKhan and Peter, 2015) and melatonin has shown anti-hypoxic ability in Chinese mitten crab (Yang et al., 2021b). Anti-stress action of melatonin on cortisol function has also been reported in fishes (Herrero et al., 2007; Azpeleta et al., 2010; Lopez-Patino et al., 2013; 2014) including zebrafish (Lunkes et al., 2021; Zhang et al., 2021). Many of its well-established physiological effects are mediated *via* high-affinity cell membrane receptors belonging to the superfamily of G-protein-coupled receptors. Membrane melatonin receptors like MT1 (MTNR1A) and MT2 (MTNR1B) are heterotrimeric G-protein-coupled receptors that interact with downstream messengers such as adenylyl cyclase, phospholipase A2 and phospholipase C (Jockers et al., 2016). MT1 and MT2 receptors are found in almost all peripheral tissues, as well as in the central nervous system and they might interact with nuclear receptors like retinoid orphan receptors and retinoid Z receptors (Jockers et al., 2016).

In view of a role for melatonin in ion homeostasis in fishes, we hypothesized that melatonin might regulate NKA functions in major ionocytes of fish and integrate its functions across major ionocytes to recover the disturbed systemic Na^+^ homeostasis in the face of hypoxia stress. To this end, we tested the regulatory and integrative actions of melatonin on NKA function in the ionocytes of non-stressed and hypoxia-stressed air-breathing fish (*Anabas testudineus* Bloch) to delineate the integrative role of melatonin in Na^+^ homeostasis and to identify how does melatonin modulate the stress and ease response in this fish. We ascertained the working of our model by measuring the melatonin concentration in plasma and the transcript abundance of mtnr1 isoforms. We analyzed transcriptional, translational and post-translational regulations of NKA functions and assessed the dynamics of NKA-immunoreactivity in ionocytes after melatonin treatment in non-stressed and immersion-stressed fish. Further, we proposed an integrative parabola model to assess the preferential action of melatonin on ionocytes and to identify the lead role of ionocyte that drives the maximum molecular regulatory mechanism of NKA function to lead recovery or ease response upon melatonin availability in hypoxia-stressed fish.

## 2 Materials and methods

### 2.1 Fish holding conditions

Adult healthy climbing perch of both sexes (*Anabas testudineus* Bloch) that belongs to order Perciformes and family Anabantidae, weighing about 35–45 g were collected from near water bodies and reared in large cement tanks. They were acclimated to laboratory conditions for 2 months in cement tanks with fresh water kept at 28 ± 1°C under natural photoperiod (12L/12D). Fish were fed with commercial fish feed daily (1% body weight) and were in the pre-spawning period (March-April). Prior to experiment, they were kept in 50 L glass aquaria (60 × 30 × 30 cm) at static conditions for 3 weeks and food was withdrawn for 24 h prior to sampling to ensure optimum experimental conditions. The regulation of Departmental Animal Ethics (DZ/10442/14) was fulfilled while rearing and experimenting the fish.

### 2.2 Experimental design

Two sets of experiments were carried out. The dose-responsive *in vivo* action of melatonin was tested in first experiment. Thirty-two laboratory-acclimated fish were grouped into four with eight fish in each group. First fish group that received injection of .65% NaCl intraperitoneally served as sham control. The remaining three groups of fish were kept for 30 min after the administration of varied doses of melatonin (12.5, 25, 50 ng g^−1^).

In second set of experiment, laboratory-acclimated fish were divided into four groups of twelve each and kept in glass tanks in triplicates (n = 144). We choose 50 ng g^−1^ of melatonin as most effective *in vivo* dose due to its ability to elicit ion transporter changes in the ionocytes of fish tissues and on the levels of thyroid hormones and cortisol in our experiment (data not presented). First and second fish groups were the non-stressed fish batch and third and fourth fish groups were the immersion-stressed fish. Fish in first group were kept for 30 min after saline injections (.65% saline) and they served as non-stressed sham control fish. Melatonin (50 ng g^−1^) was administered for 30 min to second fish group. Both third and fourth fish groups were held under wire mesh kept just below the surface water that prevented them from gulping air for 30 min. Saline injection was given to these stressed fish of third group as stressed-sham control and were kept for 30 min. Melatonin (50 ng g^−1^) was given to fourth fish group, which were also given immersion stress. All fish were handled in same way to normalize the impact of injection and sampling was done concurrently. No mortality was observed in any fish groups during the experimentation.

### 2.3 Sampling and tissue preparation

Fish in each group were netted simultaneously and anesthetized in a .1% 2-phenoxyethanol solution (SRL, Mumbai) in both experiments. Blood samples were collected from the caudal arteries using heparinized syringe and the collected blood was held on ice and analyzed for arterial blood ions. Each fish was then sacrificed by spinal transection and tissue sampling was done quickly. We excised second gill arch that represented a typical grown pair of hemibranch having well developed ionocytes, trunk kidney that holds more osmoregulatory function and anterior region of intestine just below duodenum that features enterocytes having osmoregulatory function (Abaurrea-Equisoain and Ostos-Garrido, 1996). For the analysis of NKA activity, tissues were stored in SEI (Sucrose-EDTA-Imidazole) buffer (.05 M: pH 7.1). Tissue samples from six fish of each group were also kept in incubation buffer (50 mM imidazole, 25% glycerol, .25% deoxycholate, 10 mM β-mercaptoethanol and .1 mM Na_2_EDTA) for phosphorylation-dephosphorylation studies. Tissue samples from another four fish were placed in RIPA buffer (50 mM Tris, .2 M NaCl and .1% Triton X-100, pH 7.4) for western blotting and RNA later solution (Ambion, Invitrogen) for qRT PCR immediately and kept at -80°C until analysis. For immunohistochemical analysis, two fish from each group were perfused with 4% paraformaldehyde in .1 M phosphate buffer (pH 7.2) for 15 min and then second gill arch, trunk kidney and anterior intestine were fixed in the same media for overnight at 4°C. The fixed tissues were then rinsed with .1 M phosphate buffer and placed in 25% sucrose overnight at 4°C and later stored in tissue tek (Leica Biosystems, United Kingdom) at -80°C.

#### 2.3.1 Quantification of NKA-specific activity and blood ions

The ouabain-sensitive NKA-specific activities in gill, kidney and intestinal homogenates were quantified adopting the method of Peter *et al.* (2000) modified for microplate assay (Peter et al., 2011). Saponin (.2 mg protein^−1^) was routinely added to optimize substrate accessibility. Samples in duplicates containing (1.0 µg protein) were added to a 96-well microplate containing 100 mM NaCl, 30 mM imidazole (pH 7.4), .1 mM EDTA and 5 mM MgCl_2_. .13 mM KCl was used as promoter and .14 mM ouabain was used as inhibitor. After vortexing, the assay mixture was incubated at 37°C for 15 min. The reaction was initiated by the addition of .3 mM ATP and was terminated with addition of 8.6% TCA. The liberated inorganic phosphate was measured against phosphate standard at 700 nm in Synergy HT Biotek Microplate Reader. The change in absorbance between promoter and inhibitor assays was calculated and regression analysis was employed to derive the rate of activity of NKA and expressed in µmoles Pi liberated hr^−1^ mg protein^−1^. We also quantified the total ions such as [Na^+^] [K^+^] [Cl^−^] and [Ca^2+^] levels in arterial blood collected from each fish of all groups using a gas-ion analyzer (Radiometer, ABL80 FLEX, Germany).

#### 2.3.2 Plasma melatonin

Melatonin concentrations in plasma samples were quantified based on competitive immunoenzymatic assay kits (Immunotag, United States) validated for climbing perch. Briefly, the melatonin antibody pre-coated wells were treated with 50 µL biotinylated antibody containing standards and 20 µL samples respectively. After adding 10 µL anti-MT antibody to sample wells, 50 µL strepatavidin-HRP was added. The microplate was then vortexed and incubated for 60 min at 37°C. After washing, 50 µL of substrate solution A and B were added and incubated at 37°C for 10 min in the dark. Absorbance was read at 450 nm after stopping the reaction with 1N HCl. Regression analysis was done on absorbance values and the hormone level was expressed in pg L^−1^. The intra-assay coefficient of variation was 6.2% and inter-assay coefficient of variation was 7.3%

#### 2.3.3 Transcript expression of *mtnr1* and *nkaα1* isoforms by qRT-PCR

Total RNA was isolated from the gills, kidney and anterior intestinal tissues and purified using the Pure Link RNA Mini kit (Ambion, Invitrogen, Life technologies). RNA purity was validated by optical density (OD) absorption ratio (OD 260/280 nm) using Biophotometer (Eppendorf, Biophotometer Plus) and the integrity was checked by agarose gel electrophoresis. The samples that showed a ratio between 1.8 and 2.0 were taken for cDNA synthesis. cDNA was synthesized from 1 µg of total RNA in Step-One Real Time qPCR system using the High capacity cDNA Reverse Transcription kit (Applied Biosystems, Life Technologies) following the manufacturer’s protocol. The thermal cycling conditions were as follows: 25°C for 1 min, 37°C for 120 min, 85°C for 5 min and finally 4°C holding stage. cDNA sequences of NKAα-isoforms and melatonin receptors; *nkaa1a* (JN180940), *nkaa1b* (JN180941), *nkaa1c* (JN180942), *mtnr1a* (XM-026360941.1), *mtnr1b* (XM_026357982.1), *mtnr1bb* (XM_026372231.1) and *mtnr1bb(x1x2)* (XM_026375344.1) were taken from Gen-Bank (Table 1). Primers for *nkaα1* genes were designed using Primer Express software (version 2.0.0; Applied Biosystems) and those for melatonin receptors were designed using the pick primer tool of NCBI (Table 1).

**TABLE 1:** Primer sequences of *nkaα1* subunit used in the assays.

Gene	Primer sequence (5′-3′)	Gene Bank Accession Number
*nkaα1a*	F: GCT TTC TAC TGTTGT TCT CGT C	JN180940
	R: ATC ACC AGT GCT TGT TGA G	
*nkaα1b*	F: GTA TTG TTC TTG CCA TTG TTG TC	JN180941
	R: ATC TCC ACC AAA TCT CCG A	
*nkaα1c*	F: TTT CTC CAC CAA CTG CGT	JN180942
	R: TTT GCC ACC TTC AAG ACT G	

The assay mix for *nkaα1* subunits contained the custom-made primers with FAM labeled MGB probe (Applied Biosystems, Life Technologies). The qRT-PCR was performed in duplicates using the StepOne Real Time qPCR system (Applied Biosystems) using Taqman assays (Applied Biosystems, Life Technologies). The reactions contained 5 µL of 2x Taqman universal Master mix, .5 µL 20x custom made primers (Table 1) and cDNA (1 ng) or standard (1 µL) in a total volume of 10 µL. Cycling conditions were kept as 50°C for 2 min and 95°C for 10 min for one cycle followed by 40 cycles of 95°C for 15s and 60°C for 1 min. Pre-developed eukaryotic 18S rRNA (Catalog number: 4319413E) with VIC™/MGB probe, primer limited (Accession number X03,105.1, Amplicon size: 187; Amplification Efficiency: 99.66%) was used as the endogenous control (Applied Biosystems). Data [threshold cycle (Ct) values] were collected at real time.

The assay mix for mtnr1 isoforms contained 5 µL of 2x standard SYBR Green Master Mix, 500 nM of forward or reverse primers (Table 1), cDNA (1 ng), or standard (2 µL) in a total volume of 10 µL. Cycling conditions were 50 °C for 2min and 95°C for 10min (1 cycle), followed by 40 cycles of 95 °C for 15s and 60°C for 60s. Data [threshold cycle (CT) values] were collected at each elongation step. Runs were followed by melt curve analysis of 95°C for 15s, 60°C for 60s and 95°C for 1s to confirm the presence of only a single product. β-actin and GAPDH were used as the endogenous controls (Eurofins Scientific, Germany). Data [threshold cycle (Ct) values] were collected at real time. The Ct, slope, PCR efficiency, *y*-intercept, and correlation coefficient (*R* ^2^) were calculated using the default setting of QuantStudio-3 (Applied Biosystems). The amplification efficiency for *nkaα1* subunit and mtnr were between 95%–100% and the mRNA transcript abundance was calculated by adopting 2^−ΔΔCt^ comparative threshold cycle (Ct) method (where ΔCt = ΔCt sample—ΔCt control; Livak and Schmittgen, 2001). The results were statistically analyzed using Relative Expression Software Tool 2009 (REST) and the values were then expressed relative to the control.

#### 2.3.4 Immunoblotting of NKAα protein abundance

The NKAα protein abundance in gills, kidney and intestinal tissues were quantified adopting previously described protocol for western blotting (McCormick et al., 2009) with some modifications. Briefly, tissues were homogenized in five volumes of RIPA buffer (50 mM Tris, .2 M NaCl and .1% Triton X-100; pH 7.4) containing 50 mM β-glycerophosphate, .05 mM sodium fluoride, 1 mM sodium orthovandate, .05 mM DL-dithiothreitol and .5% protease inhibitor cocktail (Sigma, United States). All tissues homogenates were centrifuged at 5,000×g for 10 min at 4°C (5430R, Eppendorf, Germany) and the supernatant of gills were centrifuged again at 20,000×g for 10 min and stored at -80°C. Protein concentrations were determined using Bradford method. Samples were thawed and then mixed with Bolt LDS sample buffer (4X) and Bolt reducing agent (10X) (Novex, United States), and were heated at 70°C for 10 min. The NKA protein abundance was quantified in gills (80 µg), kidney (40 µg) and intestinal (40 µg) homogenates using 10% SDS-polyacrylamide gel electrophoresis (Lee et al., 1998). Pre-stained protein marker (Blue Plus2 pre-stained protein standard, Novex, United States) was also loaded as a single reference lane. Following electrophoresis (Bio-Rad, United States; 80V for separating gel and 90V for stacking gel), proteins were transferred to nitrocellulose membranes using iBlot two Dry Blotting systems (Life technologies, United States). To confirm blotting efficiency, we stained the nitrocellulose membrane with Ponceau S solution (P3504, Sigma-Aldrich, United States). Nitrocellulose membranes were then blocked-in phosphate buffered saline, PBS pH 7.4 (137 mM NaCl, 3 mM KCl, 10 mM Na_2_HPO_4_ and 2 mM KH_2_PO_4_) with 5% BSA (SRL, Mumbai) for overnight at 4°C. After blocking, the membranes were rinsed with PBST (PBS containing .2% Tween 20, a detergent) and then incubated with anti-NKA α subunit (1:500; NKA α (H-300): SC-28800, Santa Cruz Biotechnology, California, United States) and anti-β-actin (1:1000; A1978, Sigma-Aldrich, United States). All primary antibodies were diluted in PBS and incubated for 1 h at room temperature. After rinsing in PBST, blots were exposed to goat anti-rabbit IgG (1:5,000; Goat anti-rabbit IgG-HRP: SC-2004, Santa Cruz Biotechnology, California, United States) and goat anti-mouse IgG (1:2000; Cat. No: 1030-05, Sourthern Biotech, Birmingham, United States) conjugated to horseradish peroxidase, diluted in PBS and incubated for 1 h at room temperature in dark. After rinsing in PBST, blots were incubated with 1:1 mixture of enhanced chemiluminescent substrates A and B (Novex invitrogen, Carlsbad, CA, United States) and the immunoreactive bands were detected using Chemidoc (Gelstan, Chennai). The band staining intensity was measured using ImageJ (Fiji, NIH, United States) and the relative immunoreactivity to the β-actin protein expression was gratified. Data were obtained from four fish in each group.

#### 2.3.5 Phosphorylation and dephosphorylation status of NKA

The phosphorylation/dephosphorylation status of NKA-rich tissue samples obtained from gills, kidney and intestine of climbing perch were assayed following the method of Ramanan and Storey (2006). Frozen tissue samples were homogenized (1:2 w/v) in homogenizing buffer containing 25 mM imidazole, pH 8.0, 10% v:v glycerol, 100 mM sucrose, 10 mM β-mercaptoethanol, .2% (w:v) sodium deoxycholate (DOC), 2 mM EDTA, 2 mM EGTA, 25 mM NaF and phenylmethylsulfonyl fluoride (PMSF) (added just before homogenization). The homogenized samples were centrifuged at 10,000×g in a cold centrifuge (5430R, Eppendorf) at 4°C for 10 min and the collected supernatant was desalted to remove endogenous ions and free phosphate using Sephadex G25 columns (PD Minitrap G-25, GE healthcare, United Kingdom). Protein content of the extracts was measured using Bradford reactions and the samples were stored at -20°C until analysis.

Sample extracts were mixed with two volumes of incubation buffer (50 mM imidazole, 25% glycerol, .25% (w/v) DOC, 10 mM β-mercaptoethanol and .1 mM EDTA) and the endogenous protein kinase activities were stimulated by the addition of 10 mM Na_2_ATP, 30 mM NaF, 50 mM MgCl_2_ and either 1) 1 mM cAMP to stimulate protein kinase A (PKA), 2) 1 mM cGMP to stimulate protein kinase G (PKG) or 3) 1.3 mM CaCl_2_ and 7 μg mL^−1^ Phorbol myristate acetate to stimulate protein kinase C (PKC). For the complete dephosphorylation of the protein, 1 IU calf intestinal alkaline phosphatase was incubated with the sample extract in incubation buffer containing 50 mM MgCl_2_ and 1.3 mM CaCl_2_. Control incubations were run along with these and contained only incubation buffer to check the sensitivity of the system. After incubation, NKA specific activity was measured in all samples using ouabain-sensitive method (Peter et al., 2000) as described earlier.

#### 2.3.6 Immunohistochemical localization of NKA*α*-rich ionocytes

Cryosections of 10 µm were made from gills, trunk kidney and anterior intestine using a cryostat (Leica CM 1850) set at -21°C. The cut was made parallel to the long axis of primary filaments of gills, but in kidney and intestine, the sections were made transversely. The sections were obtained from all fish groups and then placed on poly-L-lysine-coated slides (Sigma-Aldrich, United States), dried overnight at room temperature and stored at -80°C in airtight containers. The sections were rinsed with .1 M PBS (pH 7.2) and then with Triton added PBS (.25% Triton X-100). Endogenous peroxidase activity was blocked with 3% H_2_O_2_ in PBS and kept for 40 min at room temperature. The sections were incubated with 10% normal goat serum (GeNei, Banglore) in PBS for 1 h in humid chamber to minimize non-specific immunostaining. Sections were then exposed to anti-NKA α subunit- (NKA α (H-300): SC-28800, Santa Cruz Biotechnology, California, United States) in 1:250 dilutions in PBS and incubated overnight at 4°C. The sections were rinsed three times with Tritoned PBS and then exposed to HRP-conjugated anti-rabbit secondary antibody (Goat anti-rabbit IgG-HRP: SC-2004, Santa Cruz Biotechnology, California, United States) in 1:1000 dilutions for 1 h at room temperature. The specificity of the immunoreactivity was confirmed by running a negative control along with each section in which the incubation with primary antibody was omitted. After incubation, the slides were rinsed with Tritoned PBS for three times and the samples were stained with .06% 3, 3’ Diaminobenzidine (DAB; Sigma, United States) in .01 M Tris-HCl (pH 7.6) containing 3% H_2_O_2_ for 2 min. All the sections were then counterstained with haematoxylin, dehydrated and mounted with Distyrene Plasticizer Xylene (DPX). Adjacent kidney sections were subjected to periodic acid-Schiff (PAS) stain and counterstained with haematoxylin for the identification of proximal, distal and collecting tubules. Adjacent sections of gills and intestine were also stained with haematoxylin-eosin for the morphological identification. The mounted sections were viewed with a digital camera attached to a fluorescence microscope (Leica DM 100 LED, Germany). Photomicrographs were taken randomly at ×100 magnification for gills and kidney and ×40 magnification was made for intestine. Representative photomicrographs were taken from at least four tissue samples collected from each group fish.

#### 2.3.7 Immunofluorescence-based localization of NKA *α*-rich ionocytes

Cryostat sections stored in -80°C was brought to room temperature and were rinsed with .1 M PBS (pH 7.2) and then incubated with 1% SDS (Cat# 1610301, Bio-Rad, United States) for 4 min for the antigen retrieval (Brown et al., 1996). After incubation, the sections were washed with .1 M PBS and then with PBST (.25% Triton X-100) for 10 min. The sections were then incubated with 10% antibody dilution buffer (ADB) containing 10% normal goat serum (GeNei, Banglore), 3% bovine serum albumin (SRL, Mumbai) and .05% Triton X-100 for 30 min. Due to the discontinuation of SC-28800, we purchased mouse monoclonal NKAα1 subunit (a6F) from Developmental Studies Hybridoma Bank, Iowa city, United States diluted 1:10 in ADB and was applied to sections and kept at 4°C overnight in humid chamber. The sections were rinsed three times with PBST and then exposed to Alexa-Fluor 488 labeled goat polyclonal anti-mouse secondary antibody (1:200 dilution, Cat# Ab150113, Abcam, United States) for 2 h at room temperature. The sections were then rinsed with PBS three times for 15 min and incubated with 1 µM 4’, 6-diamidino -2- phenylindole, dihydrochloride (DAPI, Cat# D9542, Sigma-Aldrich, United States) for 1 min and later rinsed in PBS. Sections were mounted on Fluoromount Aqueous mounting medium (Cat# F4680, Sigma-Aldrich, United States). The specificity of the immunoreaction was confirmed by running a negative control along with each assay in which the incubation with primary antibody was omitted. Representative fluorescence images were captured (n = 4) using a Confocal Laser Scanning Microscope (Leica TCS SP8, Leica Microsystems, Germany) with a Leica Application Suite X (LAS X) software. The staining intensity (mean grayscale/pixel) of immunoreactive cells from each group was measured using ImageJ (National Institute of Health, United States). A single threshold level for each image was used to quantify immunoreactive regions. Background staining intensity value of negative control was subtracted from each staining intensity value and the difference between groups evaluated statistically and considered the control as 100%.

#### 2.3.8 Integrative parabola modeling for assessing the differential recovery response of melatonin in ionocytes of hypoxia-stressed fish

An integrative parabola model was developed to represent integrative and preferential action of melatonin on NKA-driven recovery response that exists in tested ionocytes in test species. This model utilizes the response pattern of tested variables of NKA regulation in gills, kidney and intestine of climbing perch under non-stressed, immersion-stressed and immersion-stressed plus melatonin treatment. First, we illustrated a schematic of NKA-driven recovery response to exogenous melatonin. A typical stress response curve was first generated to show how a tested variable in a fish tissue respond to 30min of immersion stress, compared to non-stressed fish. These were plotted on *X*-axis. When exogenous melatonin was given to immersion-stressed fish, a typical recovery curve in the form of parabola was obtained and that represents a typical recovery response. subsequently, varied patterns of recovery response curve were obtained for each tested parameter that differs from a typical parabola of melatonin-induced recovery response. By using multi Y-line graphs in Origin 2018 software (Origin Lab, United States), we plotted the quantified magnitude of changes that produced for NKA functions such as NKA specific activity, isoform *nkaα1a*, *nkaα1b and nkaα1c* mRNA expression*,* NKAα protein abundance and immunofluorescence staining intensity. The integrative parabola model that generated subsequent measurement of parabola thus provided a qualitative model for the assessment of recovery response of osmoregulatory epithelia to melatonin in stressed fish.

### 2.4 Statistical analysis

Data were collected from all experimental fish in each group including the analysis of NKA-specific assay and phosphorylation/dephosphorylation assays. Normal distribution and variance homogeneity of the data were checked before statistical analysis. One-way analysis of variance (ANOVA) followed by SNK comparison test was used for finding significance between treatments. Each value is represented as mean ± SEM. Significance between the non-stressed control Vs. melatonin-treated fish or immersion stressed fish (’*‘), stressed fish Vs. stressed plus melatonin-treated fish (’#‘) and non-stressed control Vs. stressed melatonin-treated fish (’@‘) were analyzed with the help of Graphpad Software (Graphpad Instat-3, San Diego) and the level of significance was accepted (*p* < .05) and graphs were plotted using Sigmaplot 11.0. The relative mRNA expression of isoforms was statistically analyzed using Relative Expression Software Tool 2009 (REST) and the significant difference between fish groups were recorded after depicting significance at *p* < .05.

## 3 Results

### 3.1 Effect of melatonin treatment on plasma melatonin concentration in non-stressed and hypoxia-stressed fish

**FIGURE 1:**
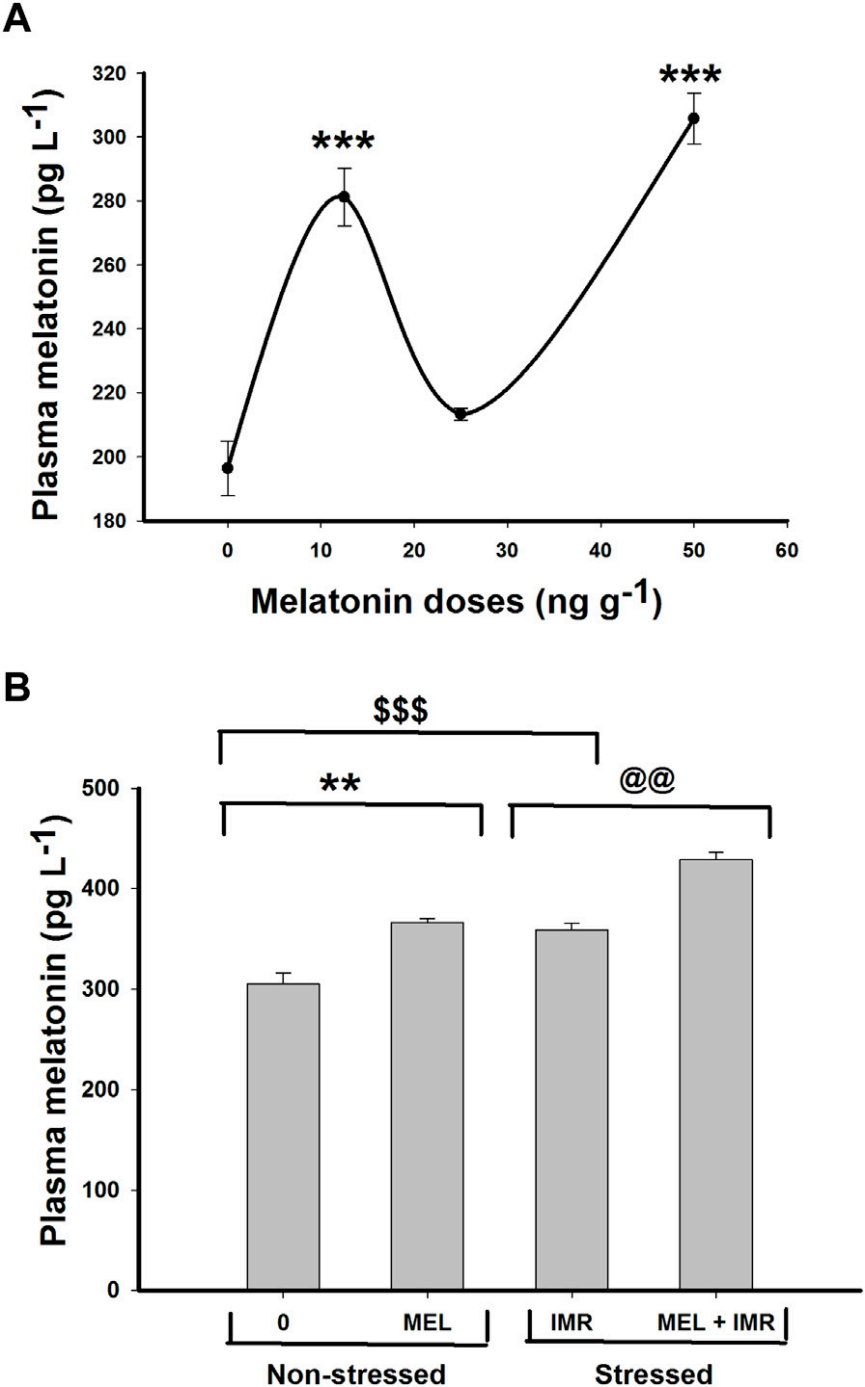
*In vivo* action of melatonin treatment for 30 min on plasma melatonin concentration in non-stressed or immersion-stressed air-breathing fish. **(A)** shows dose-responsive action of melatonin (MEL; 12.5, 25 and 50 ng g^−1^) and **(B)** represents the action of melatonin (MEL; 50 ng g^−1^) for 30 min in immersion-stressed fish (IMR). Each plot represents mean ± SE.

The plasma melatonin concentration significantly increased (*p* < .001) after low and high doses of melatonin treatment (Figure 1A). Likewise, a significant rise in plasma melatonin concentration (*p* < .01) occurred after melatonin treatment in both non-stressed and hypoxia-stressed fish (Figure 1B). Interestingly, the plasma melatonin concentration increased significantly after 30 min of forced immersion (Figure 1B) that showed a substantial hypoxemia in these fish as reported earlier (Peter and Gayathry, 2021).

### 3.2 Dose-responsive *in vivo* action of melatonin on NKA activity in ionocyte epithelia and blood ions in non-stressed fish

**FIGURE 2:**
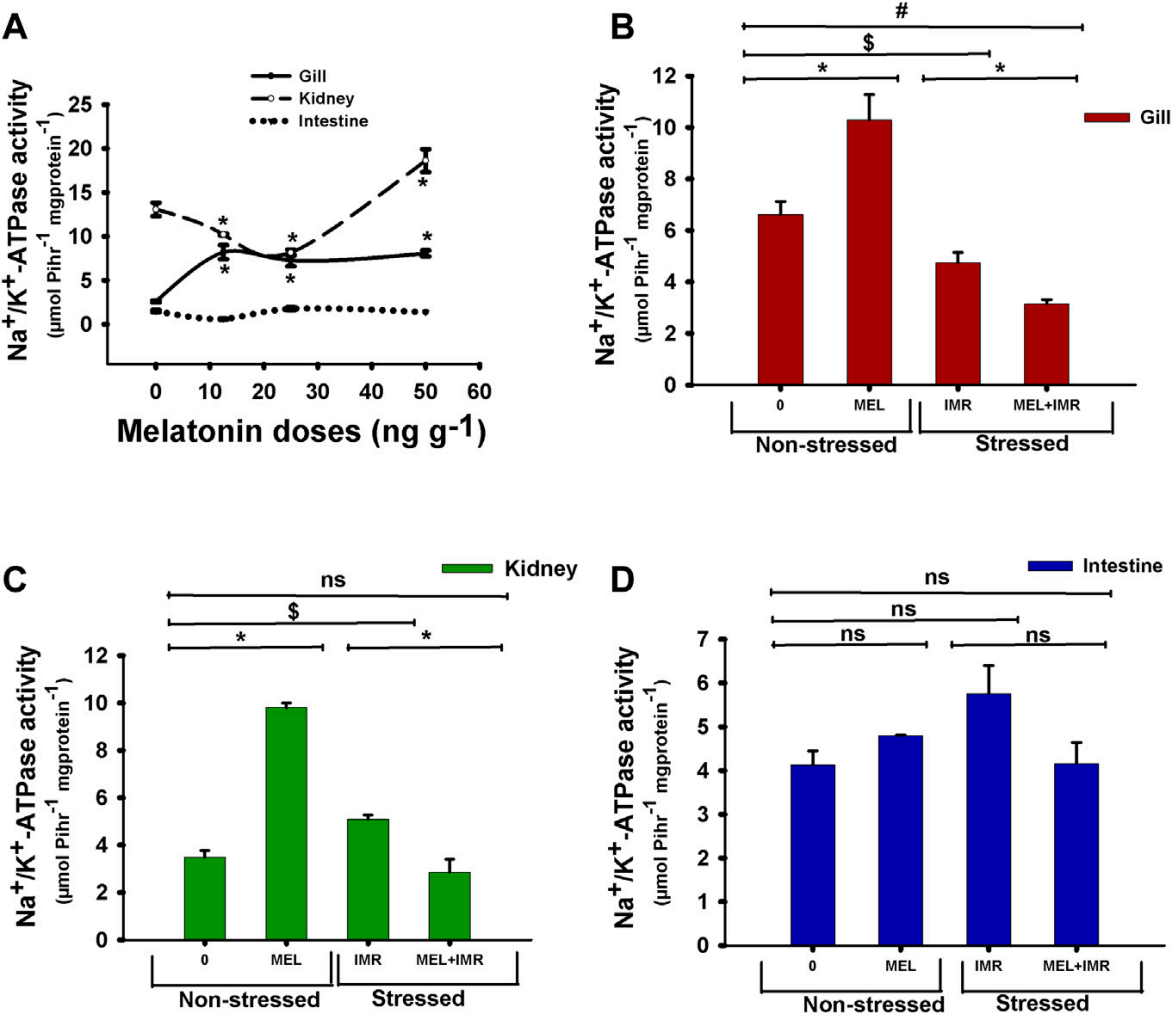
*In vivo* action of melatonin treatment on Na^+^/K^+^-ATPase (NKA) activity in gills, kidney and intestine of non-stressed or immersion-stressed air-breathing fish. Figure 2A shows dose-responsive action of melatonin (MEL; 12.5, 25 and 50 ng g^−1^) and Figures 2B–D represent the action of melatonin (MEL; 50 ng g^−1^) on the activity pattern of NKA in gills **(B)**, kidney **(C)** and intestine **(D)** in non-stressed or immersion-stressed fish (Figures 2B–D). For Figure 2A the significance, accepted at *p* < .05 level, are represented as “*” compared to control fish (0 ng g^−1^). For Figures 2B–D “*” represent significance between non-stressed control (0) and melatonin (MEL) treated or comparison between IMR and MEL + IMR fish. “$” represents significance between non-stressed control (0) and IMR fish. “#” denotes significance between non-stressed control (0) and MEL + IMR fish.

Short-term administration of melatonin at doses (12.5, 25, 50 ng g^−1^) significantly increased the ouabain-sensitive NKA-specific activity in the branchial epithelia of climbing perch compared to control fish (Figure 2A). On the contrary, low doses of melatonin treatment significantly decreased the NKA activity in the kidney (Figure 2A), whereas 50 ng g^−1^ melatonin increased the kidney NKA activity (Figure 2A). However, melatonin treatment did not produce any modification on NKA activity in intestine (Figure 2A). We chose 50 ng g^−1^ melatonin dose for our further study to test its action in hypoxia-stressed fish as that dose showed substantial response to the tested variables. The analysis of total ion contents in blood showed dose-responsive effects of melatonin treatment in non-stressed fish (Table 2). Significant fall in [K^+^] and [Cl^−^] contents and significant dose-responsive rise in [nCa^2+^] content were found after melatonin treatment (Table 2).

**TABLE 2:** Dose-responsive *in vivo* action of melatonin (0, 12.5, 25, 50 ng g^−1^) injection for 30 min on blood ions in non-stressed *A. testudineus*.

Arterial blood (mmol L^−1^)	Melatonin (ng g^−1^)			
	0	12.5	25	50
[Na^+^]	155.3 ± 0.80	156.0 ± 0.41	155.0 ± 0.84	155.40 ± 0.25
[K^+^]	7.0 ± 0.21	5.3 ± 0.21*	4.4 ± 0.23*	4.1 ± 0.27*
[Cl^−^]	126.0 ± 0.71	116.0 ± 0.97*	116.8 ± 1.46*	117.4 ± 1.17*
[nCa^2+^]	0.56 ± 0.04	1.18 ± 0.07*	1.09 ± 0.02*	1.26 ± 0.08**

Each value is mean ± SEM, of eight fish.

^*^(*p* < 0.05) denotes sinificant difference between control and melatonin-treated fish.

### 3.3 Melatonin action on NKA activity and blood ions in hypoxia-stressed fish

The NKA activity in the gills of melatonin-treated fish increased significantly in non-stressed fish (Figure 2B). But melatonin treatment favored a decline in NKA activity in the gills of immersion-stressed fish which showed a declined NKA activity in hypoxia condition (Figure 2B). Similarly, NKA-specific activity in kidney showed a significant rise after melatonin treatment in non-stressed fish, whereas melatonin favored the declined activity in immersion-stressed fish which also showed a declined NKA activity in hypoxic state (Figure 2C). In contrast, neither melatonin nor hypoxia produced any modification in the intestinal NKA activity (Figure 2D). Furthermore, the intestinal NKA activity remained unaffected after melatonin treatment in immersion-stressed fish (Figure 2D).

Melatonin treatment produced a fall in [K^+^] content in blood of non-stressed fish but not [Na^+^] content (Table 3). Significant decrease in [Na^+^] and [K^+^] contents were found in immersion-stressed fish (Table 3). Interestingly, melatonin treatment could recover the loss of [Na^+^] and the loss of [K^+^] contents in immersion-stressed fish (Table 3). However, a significant rise in [nCa^2+^] level occurred after melatonin treatment in stressed fish, compared to non-stressed control fish (Table 3).

**TABLE 3:** Action of melatonin (50 ng g^−1^) for 30 min on blood ions in non-stressed and immersion-stressed *A*. *testudineus*.

Blood ions (mmol L^−1^)	*Non-stressed*		*Stressed*	
	Control	Melatonin	Immersion	Melatonin +Immersion
[Na^+^]	152.8 ± 0.98	153.6 ± 0.95	146.00 ± 1.73^#^	155.0 ± 1.27^@^
[K^+^]	5.8 ± 0.21	4.4 ± 0.14*	4.6 ± 0.22^#^	6.3 ± 0.27^@^
[Cl^−^]	114.3 ± 0.87	115.3 ± 0.57	118.80 ± 3.09	113.63 ± 0.91
[nCa^2+^]	0.92 ± 0.01	1.24 ± 0.07*	1.20 ± 0.05^#^	1.32 ± 0.06^$^

Each value is mean ± SEM, of eight fish.

*(*p* < 0.05) denotes sinificance between non-stressed control and melatonin treated fish.

#(*p* < 0.05) denotes sinificance between non-stressed control and immersion-stressed fish.

a(*p* < 0.05) denotes sinificance between immersion-stressed fish and melatonin-treated plus immersion-stressed fish.

$(*p* < 0.05) denotes sinificance between non-stressed control fish and melatonin-treated plus immersion-stressed fish.

### 3.4 Melatonin action on mRNA expression of *nkaα1* isoforms in non-stressed and hypoxia-stressed fish

**FIGURE 3:**
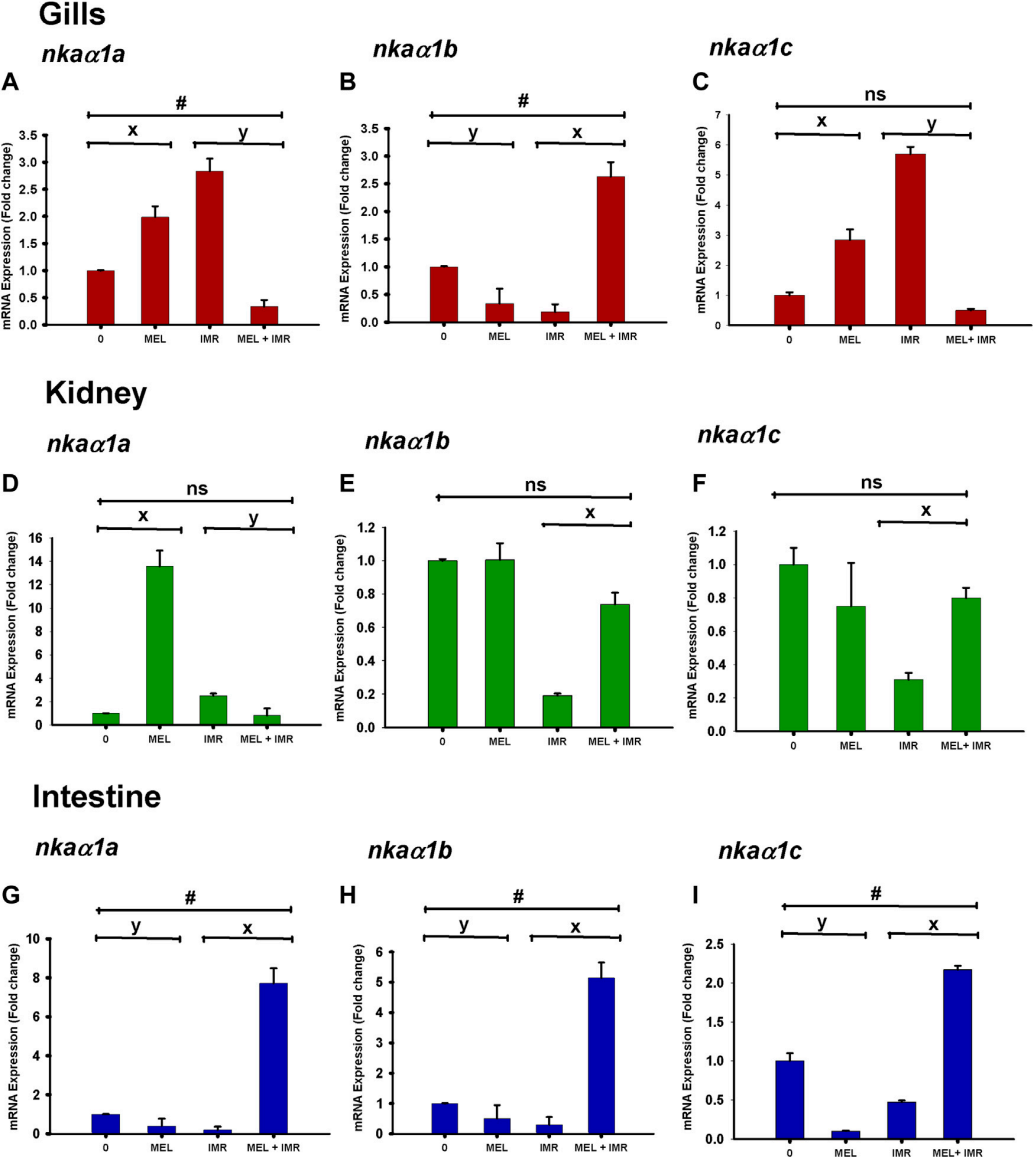
Relative mRNA expression of NKAα1-subunit isoform *nkaα1a* **(A,D,G)**, *nkaα1b* **(B,E,H)** and *nkaα1c* **(C,F,I)** in non-stressed and immersion stressed fish after melatonin-treatment (MEL; 50 ng g^−1^). 18S rRNA was used as the endogenous control. The data obtained from each group were statistically analyzed using Relative Expression Software Tool 2009 (REST) and the significant levels are represented. “*” represents significance between non-stressed control (0) and melatonin (MEL) treated or comparison between IMR and MEL + IMR fish. “#” denotes significance between non-stressed control (0) and MEL + IMR fish.

Differential and spatial regulation of *nkaα1a, nkaα1b* and *nkaα1c* isoform expression were found after melatonin treatment in gill, kidney and intestinal epithelia of non-stressed fish. The mRNA expression of *nkaα1a* isoform showed upregulation after melatonin treatment in gills and kidney of non-stressed fish (Figures 3A, D). In constrast, the expression of this isoform downregulated in these tissues after melatonin-treatment in stressed fish (Figures 3A, D). A downregulation of *nkaα1b* isoform was found after melatonin treatment in gills and intestine but not in kidney of non-stressed fish (Figures 3B, E, H). On the contrary, melatonin treatment upregulated the *nkaα1b* isoform expression in all tissues of stressed fish (Figures 3B, E, H). Interestingly, the *nkaα1a, nkaα1b* and *nkaα1c* isoforms downregulated after melatonin treatment in intestine of non-stressed fish, but it showed an upregulation after melatonin treatment in immersion stressed fish (Figures 3G–I). The mRNA expression of the *nkaα1c* isoform showed downregulation after melatonin treatment in kidney of non-stressed fish (Figure 3F). In constrast, the expression of isoform upregulated in this tissue after melatonin-treatment in stressed fish (Figure 3F). The *nkaα1c* isoform upregulated in gills after melatonin treatment in non-stressed fish, but it showed a downregulation after melatonin treatment in immersion stressed fish (Figure 3C).

### 3.5 Melatonin action on mRNA expression of melatonin receptors in non-stressed and hypoxia-stressed fish

**FIGURE 4:**
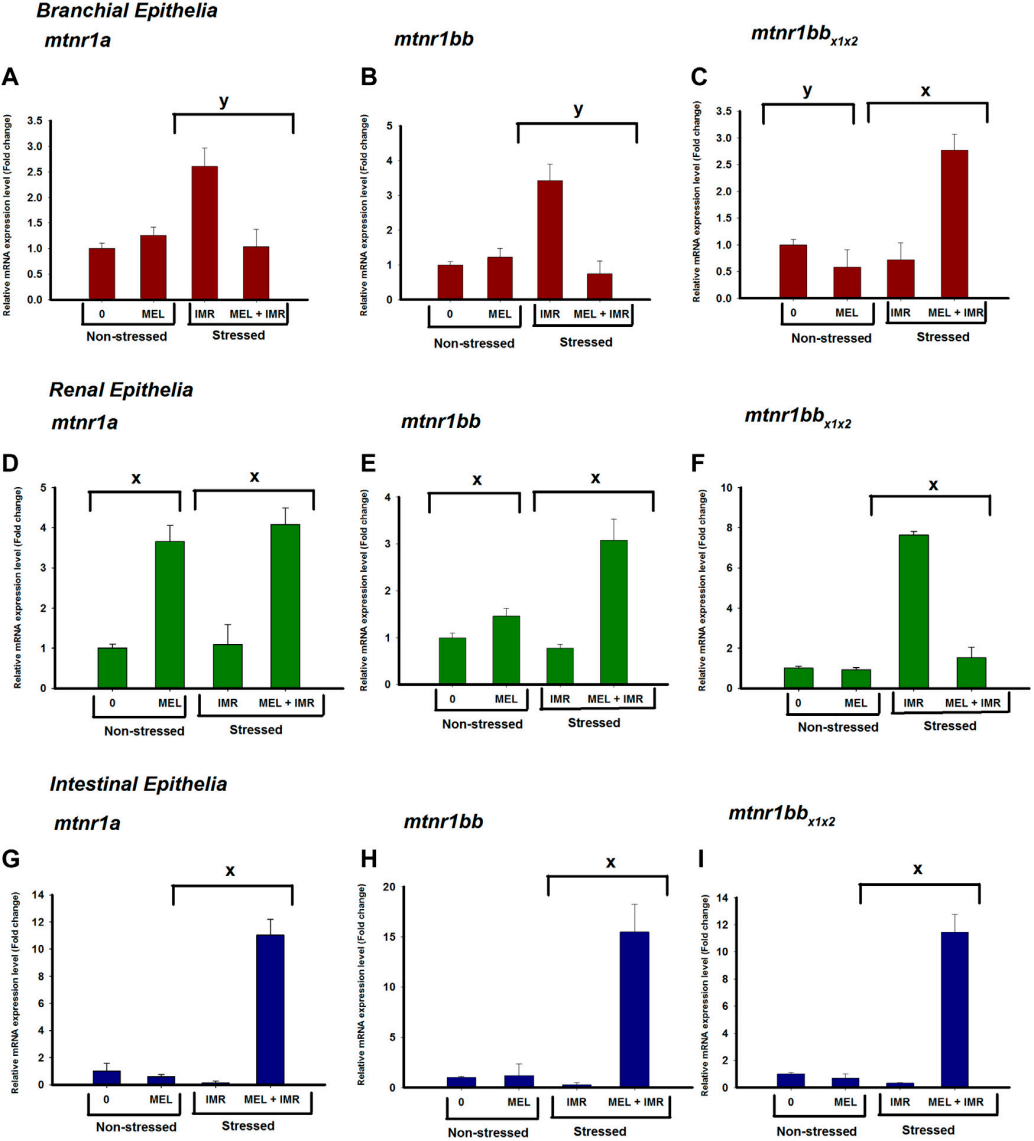
Relative mRNA expression of *mtnr1* subunit isoforms; *mtnr1a* **(A,D,G)**, *mtnr1bb* **(B,E,H)** and *mtnr1bbx1x22* **(C,F,I)** in non-stressed and immersion-stressed fish after melatonin-treatment (MEL; 50 ng g^−1^) for 30 min 18S rRNA was used as the endogenous control. The data obtained were statistically analyzed using Relative Expression Software Tool 2009 (REST) and the significant levels are represented. “*” represents significance between non-stressed control (0) and melatonin (MEL) treated or comparison between IMR and MEL + IMR fish. “#” denotes significance between non-stressed control (0) and MEL + IMR fish.

The mRNA expression of *mtnr1a* and *mtnr1bb* in gills significantly downregulated after melatonin treatment in hypoxia-stressed fish, but it showed an upregulation in intestine (Figure 4). The mRNA expression of *mtnr1a* and *mtnr1bb* significantly upregulated in kidney after melatonin treatment in both non-stressed and hypoxia-stressed fish (Figure 4). Melatonin treatment significantly unregulated the *mtnr1b* mRNA expression in the intestine of both non-stressed and hypoxia-stressed fish (Figure 4). But, melatonin treatment significantly downregulated the mRNA expression of *mtnr1bb* variant in the gills of non-stressed fish, but it significantly upregulated its expression in hypoxia-stressed fish (Figure 4). The mRNA expression of *mtnr1bb* variant significantly dowregulated after melatonin treatment in the kidney of hypoxia-stressed fish (Figure 4).

### 3.6 Melatonin action on NKAα-subunit protein abundance in non-stressed and hypoxia-stressed fish

**FIGURE 5:**
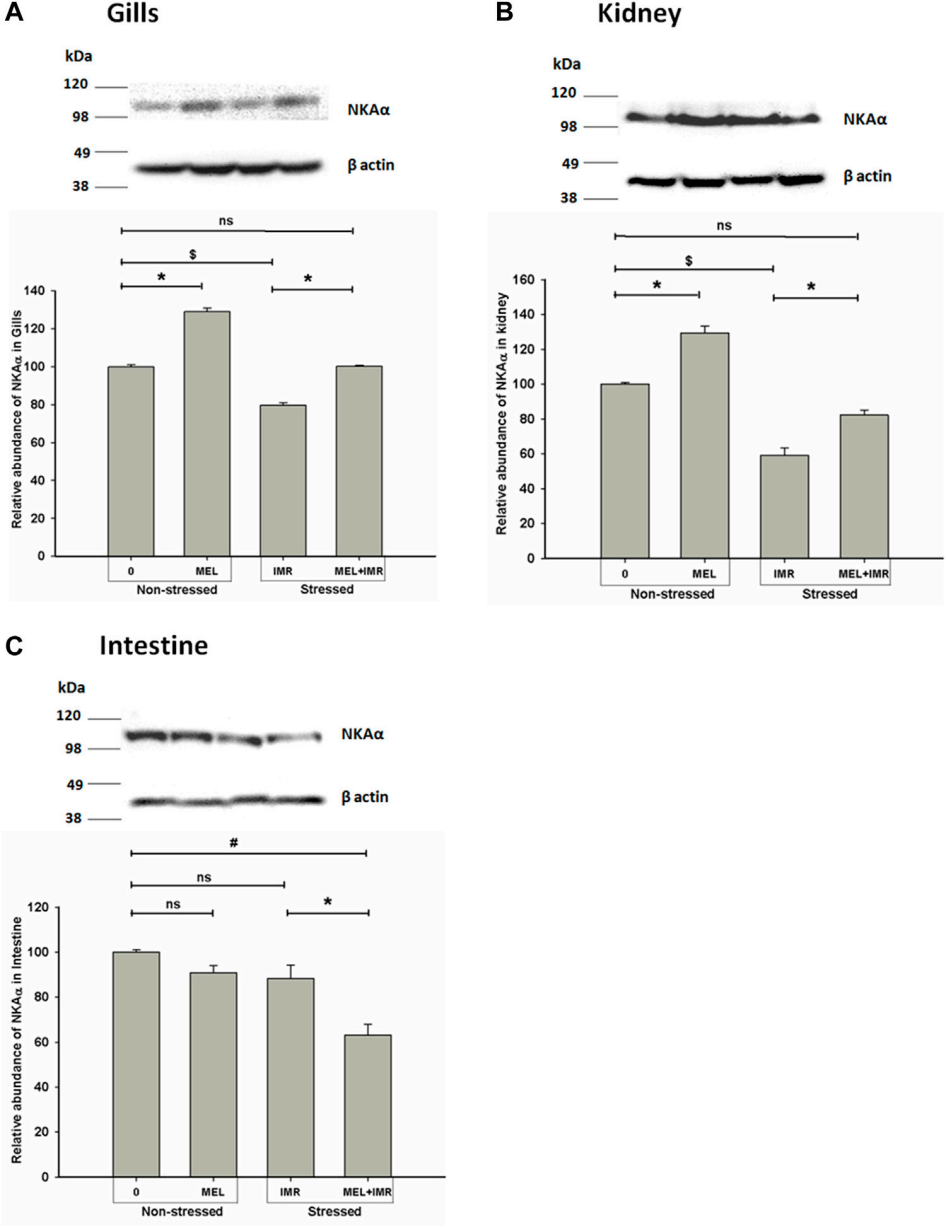
Representative western blots showing immunoblotting of NKAα subunit protein abundance in gills **(A)**, kidney **(B)** and intestine **(C)** after *in vivo* melatonin (50 ng g^−1^) treatment (Mel in non-stressed and immersion-stressed fish (IMR). β-actin was used as an internal control for the densitometric analysis. Protein was harvested from each group and the significance between groups was accepted at *p* < .05 level. “*” represents significance between non-stressed control (0) and melatonin (MEL) treated or comparison between IMR and MEL + IMR fish. “#” denotes significance between non-stressed control (0) and MEL + IMR fish.

We quantified the NKA**α**-protein density in osmoregulatory epithelia of perch in non-stressed and stressed conditions and found that translational regulation of NKAα subunit occurred after melatonin treatment in these fish. Western blot analysis of protein isolated from these tissues provided single NKA-immunoreactive band at ∼110 kDa. In non-stressed fish, the relative abundance of NKA α protein in branchial epithelia increased significantly after melatonin treatment (Figure 5A). But induction of immersion-stress significantly decreased the immunoreactivity when compared to non-stressed control (data not shown) and the melatonin treatment produced a significant rise in NKAα-protein abundance in this tissue of stressed fish (Figure 5A). Similarly, the NKAα protein density increased substantially after melatonin treatment in non-stressed renal epithelia of melatonin-treated fish (Figure 5B). A significant rise in NKAα protein density after melatonin treatment was found in the renal tissues of immersion-stressed fish (Figure 5B). Compared to the non-stressed sham fish, the melatonin treatment exhibited little influence on NKAα protein expression in intestinal epithelia (Figure 5C). In constrast, melatonin treatment lowered the protein abundance significantly in immersion-stressed fish (Figure 5C).

### 3.7 Melatonin action on NKA phosphorylation/dephosphorylation status in osmoregulatory epithelia of non-stressed and hypoxia-stressed fish

**FIGURE 6:**
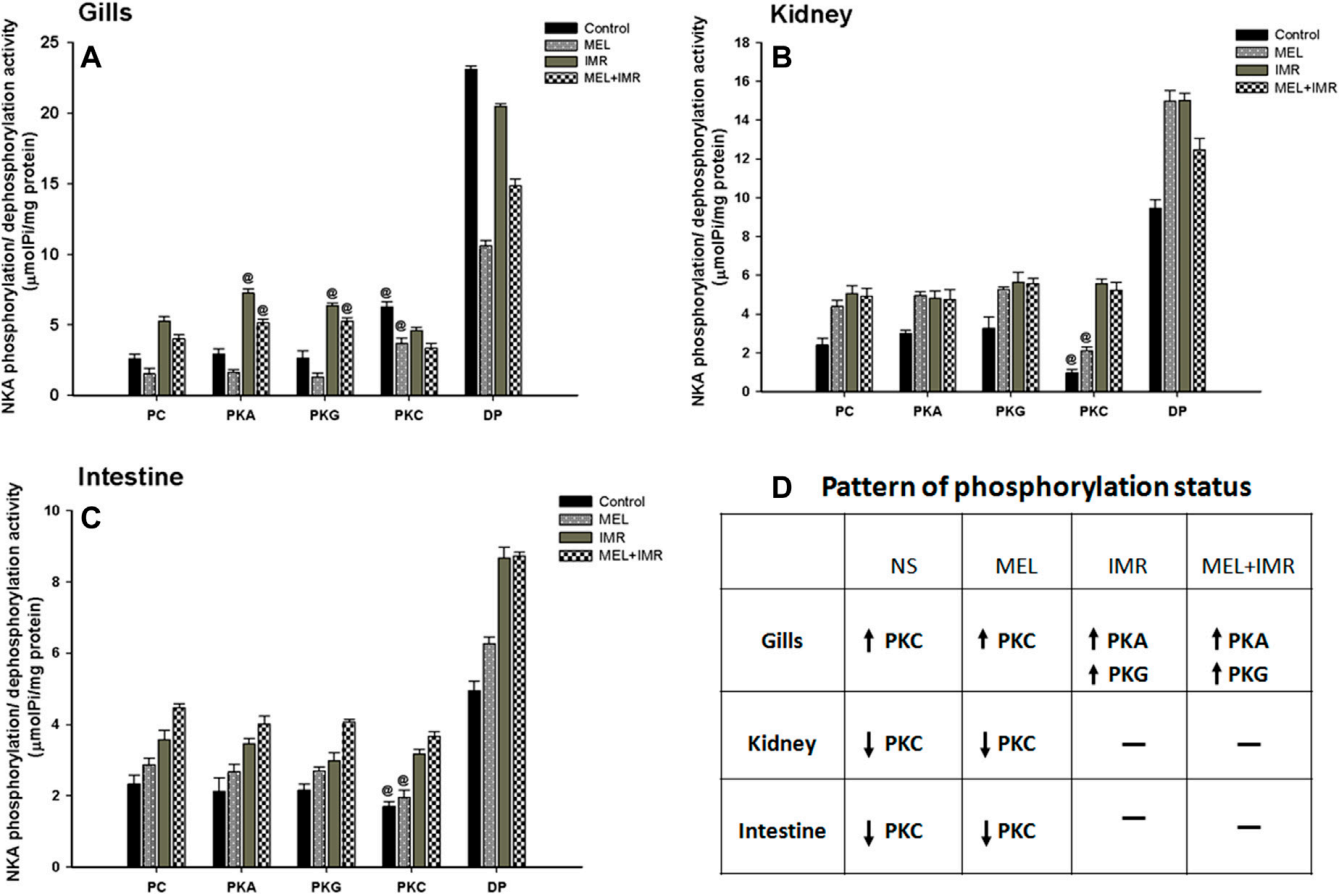
Phosphorylation and dephosphorylation status of NKA in gill epithelia **(A)**, posterior kidney **(B)** and anterior intestine **(C)** after melatonin (Mel) treatment in non-stressed or immersion-stressed (IMR) fish. PC, Phosphorylated control; PKA, Protein kinase A; PKG, Protein kinase G; PKC, Protein kinase C; DP, dephosphorylated control using alkaline phosphatase. Each column represents mean ± SEM and the significance were accepted when *p* < .05. “@” denotes significance between non-stressed and stressed fish. “#” denotes significance between non-stressed control (0) and immersion stressed (IMR) + melatonin (MEL) treated fish.

Phosphorylation of NKA by protein kinases has been identified as short-term modulators of NKA function. We tested how phosphorylation/dephosphorylation by protein kinases regulates NKA activity in response to melatonin treatments in non-stressed and immersion-stressed conditions. Activation of PKC-dependent phosphorylation was found in branchial epithelia of non-stressed and melatonin-treated fish (Figures 6A, D). In constrast, deactivation of PKC-dependent phosphorylation occurred in renal and intestinal epithelia of non-stressed and melatonin-treated fish (Figures 6B–D). Unexpectedly, activation of both PKA and PKG-dependent phosphorylation occurred in gills of stressed fish and in stressed fish that received melatonin (Figures 6A, D). These responses were, however, not found in renal and intestinal epithelia (Figures 6B–D). A complete dephosphorylation of NKA by alkaline phosphatase demonstrated maximum activity of NKA in all these tested tissues (Figures 6A–C).

### 3.8 Immunohistochemical and immunofluorescence-based NKAα-immunoreactivity in hypoxia-stressed ionocytes

**FIGURE 7:**
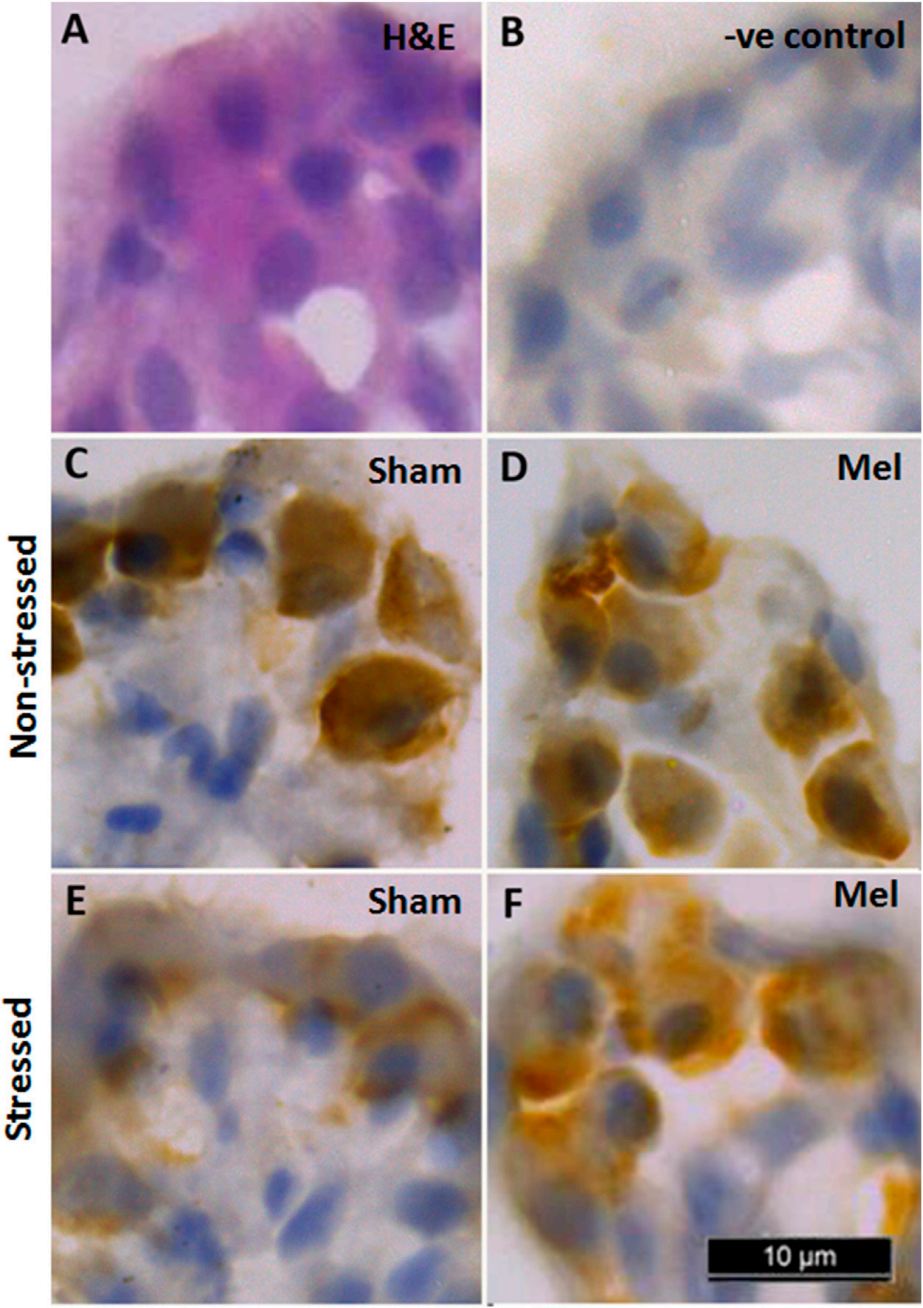
Representative immunohistochemical images of NKA-rich branchial ionocytes immunolocalized with NKAα-antibodies in the gill filaments of air-breathing fish. Cryostat sections of gill filaments showed branchial ionocytes located on the edges of reduced secondary lamellae as semicircular ring [H&E stained; **(A)**. Varied pattern of NKA-immunoreactivity was found in gill epithelia of non-stressed fish **(C)**, melatonin-treated **(D)**; Mel] and immersion-stressed fish (E; IMR). Melatonin (50 ng g^−1^) was given to immersion stressed fish **(F)**. Negative control sections were kept under the same conditions without primary antibody and that yielded no immunoreactivity **(B)**. Scale bar = 10 μm.

The distribution pattern of branchial ionocytes has been described previously in the gill epithelia of climbing perch (Peter et al., 2011). In this fish, branchial ionocytes are mainly located peripherally on the secondary lamellae as round or elliptical cell with a round nucleus at the base. The secondary lamellae are reduced as this fish breathes atmospheric air and possess labyrinth type accessory respiratory organs. Both immunohistochemistry and immunofluorescence images of branchial epithelia showed that the NKA*α*-immunoreactivity was concentrated on these ionocytes basolaterally, a typical indicator of branchial ionocytes (Figures 7, 10). Melatonin treatment produced substantial NKA*α*-immunoreactivity to branchial ionocytes (Figures 7, 10), which was evident more on basolateral sides of branchial ionocytes. Immersion-stress, however, produced a lowered NKA-immunoreactivity to the branchial ionocytes (Figures 7, 10). However, melatonin treatment produced more intense NKA-immunoreactivity in the branchial ionocytes of stressed fish (Figures 7, 10).

**FIGURE 8:**
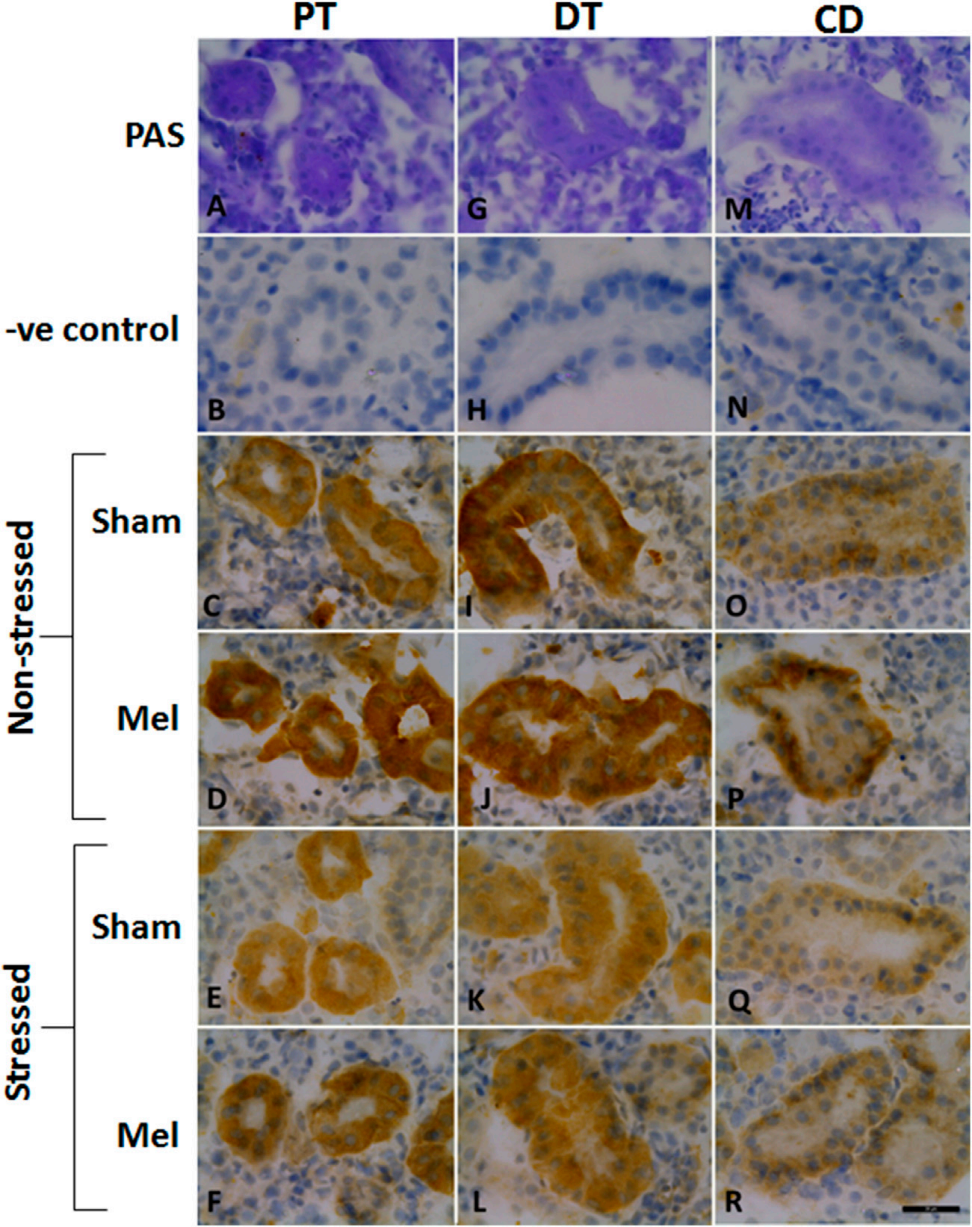
Representative immunohistochemical images of the proximal kidney tubules **(A–F)**, distal kidney tubules **(G–L)** and collecting ducts **(M–R)** of climbing perch showing renal ionocytes in melatonin (Mel) treated or immersion-stressed (IMR) or both. Adjacent cryostat sections were stained with Periodic acid-Schiff-hematoxylin stain for distinguishing the different segments of tubules **(A,G,M)** and later immunostained with anti-Na^+^/K^+^ATPase α-antibody. Images of non-stressed fish control **(C,I,O)**, melatonin [50 ng g^−1^; **(D, J, P)**] and immersion-stressed fish control **(E,K,Q)**. Melatonin was given to immersion stressed fish **(F,L,R)**. Negative controls were kept under the same conditions without primary antibody that yielded no immunoreactivity **(B,H,N)**. Scale bar = 20 μm.

Structurally distinct NKA*α*-immunostaining was found in the discrete regions of the renal tubules of climbing perch. Specific NKA *α*-immunoreactivity was obtained in the renal epithelial cells localized in the renal tubules, whereas such immunoreactivity was absent in glomeruli (data not shown). The different segments of renal tubules were distinguished with the help of PAS staining (Teranishi and Kaneko, 2010). The proximal tubules possess a single layer of columnar epithelial cells that are equipped with PAS-positive brush boarder at the apical membrane (Figure 8A). The distal renal tubular segment has a single layer of cuboidal cells with a centrally located nucleus but lack apical brush boarder (Figure 8G). On the contrary, the collecting renal tubules have a single layer of columnar epithelial cells with a centrally located nucleus and surrounded by connective tissues (Figure 8M). Specific NKA*α*-immunoreactivity and morphologically distinct ionocyte distribution have been found in proximal, distal and collecting tubules (Figures 8C, I, O; Figure 11) as subtypes of renal ionocytes. The immunoreactivity in the immunohistochemical and immunofluorescence images showed more intense and was strongly localized in the entire cells of distal tubules, whereas the immunoreactivity was restricted to the basolateral membrane in the proximal and collecting tubules. The pattern of immunohistochemical NKA*α*-immunoreactivity in these renal ionocyte subtypes namely; proximal, distal and collecting tubular ionocytes of non-stressed climbing perch showed intense immunoreactivity to melatonin-treatment (Figures 8D, J, P). The immunofluorescence images also showed a similar pattern of immunoreactivity to melatonin treatment (Figure 11). Evidence for a recovery of NKA*α*-immunoreactivity after melatonin treatment was found in the proximal and distal tubular ionocytes of immersion-stressed fish (Figures 8F, L; Figure 11). However, the distribution pattern of NKA*α-*immunoreactivity was lesser in the collecting tubule of immersion-stressed fish than the immunoreactivity in the ionocytes of proximal and distal tubules (Figure 8Q). Similarly, melatonin treatment has little effect on the immunoreactivity of these ionocytes in immersion-stressed fish that received melatonin treatment (Figures 8R, 11).

**FIGURE 9:**
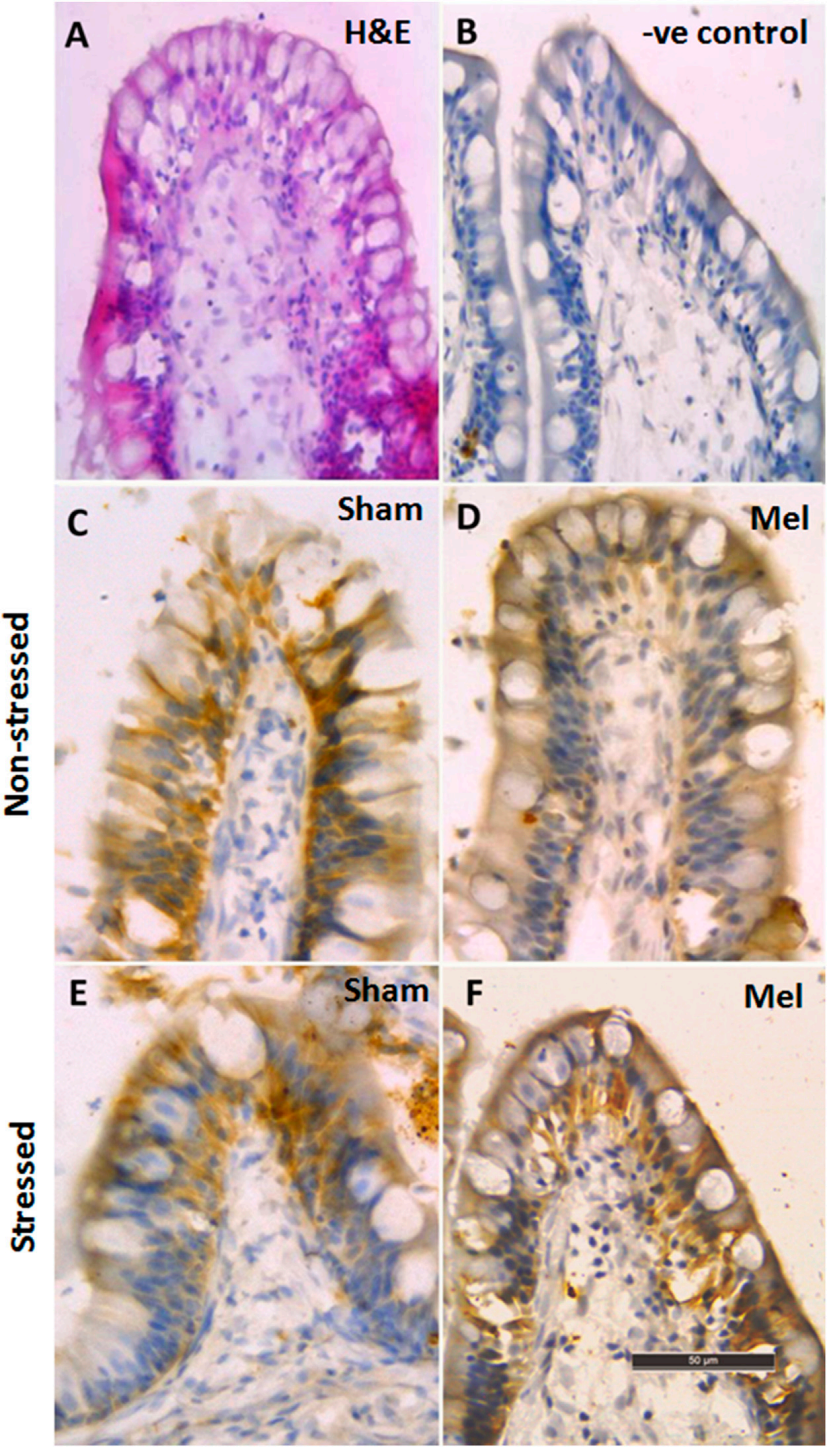
Immunohistochemical photomicrographs of intestinal villi in climbing perch showing immunoreactivity of NKAα-antibody. Cryostat sections of intestinal villi showing the distribution pattern of enteric ionocytes by H&E staining **(A)** in non-stressed fish control **(C)** and immersion-stressed fish control **(E)**. Melatonin (50 ng g^−1^; Mel) was given to non-stressed **(D)** and immersion stressed **(F)**; IMR) fish. Negative controls were kept under the same conditions without primary antibody that yielded no immunoreactivity **(B)**. Scale bar = 50 μm.

Immunohistochemical and immunofluorescence analyses of ionocytes in the anterior intestinal epithelia of climbing perch revealed a prominent NKA*α* immunostaining in basolateral regions of columnar cells in the intestinal villi and these enteric ionocytes were extended to the base of epithelial cells (Figures 9, 12). In contrast, we found an aggregated distribution and prominent intensity of NKA*α*-immunoreactivity in both apical and basel regions of the enteric ionocytes in the immersion-stressed fish (Figures 9E; Figure 12). However, the NKA*α*-immunoreactivity was less in the enteric ionocytes of both non-stressed fish and immersion-stressed fish after melatonin treatment (Figures 7D, F; Figure 12).

**FIGURE 10:**
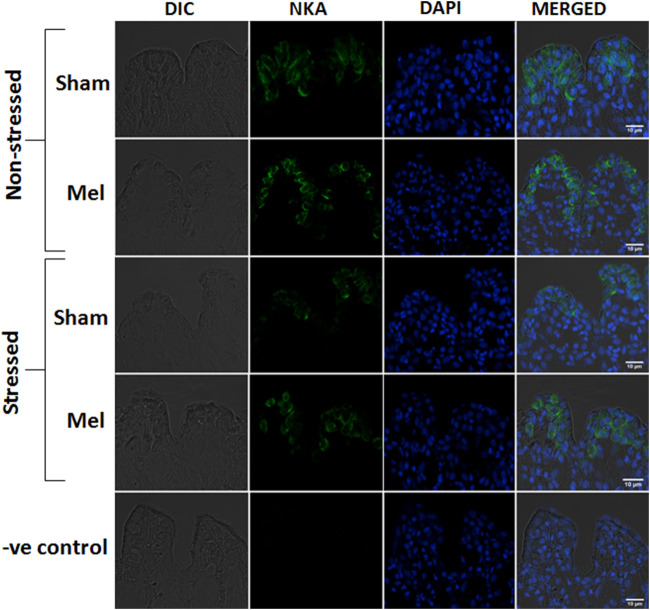
Confocal images of immunofluorescence staining of NKAα-subunit in the secondary lamellae of gills after melatonin (Mel) treatment in non-stressed and immersion stressed (IMR) climbing perch. Gill filaments were incubated with mouse monoclonal Na^+^/K^+^ATPase α1 antibody (green) and nuclear stain DAPI (blue). Branchial ionocytes with rich NKAα1-immunoreactivity are seen located in the secondary lamellae and their distribution pattern and intensity are modified after melatonin treatment in non-stressed and immersion-stressed fish. Each panel starts with DIC (differential interference contrast) image, fluorescence images of NKAα1 (green) and DAPI (blue) and a merged image in DIC. The last panel shows the corresponding images of negative control in which the incubation with primary antibody was omitted. Scale bar 10 μm.

**FIGURE 11:**
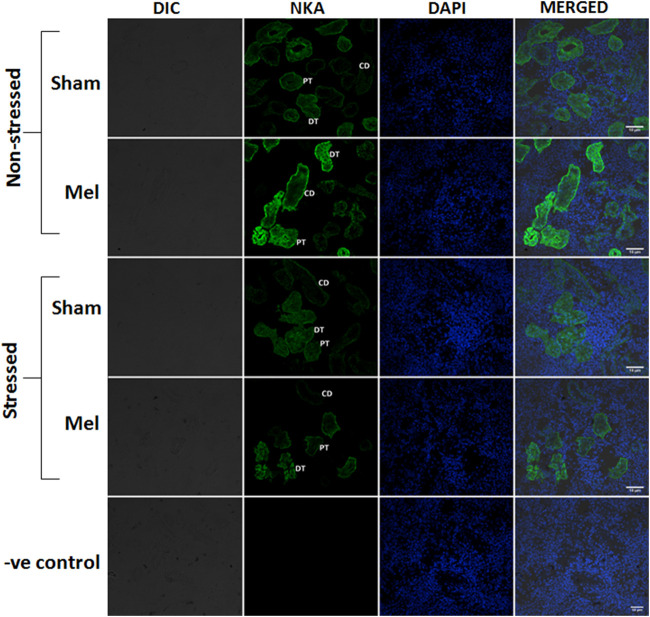
Confocal images showing the distribution pattern of NKAα1 immunoreactivity in the different regions of tubules namely: proximal tubule (PT), distal tubule (DT) and collecting duct (CD) in melatonin-treated (Mel) non-stressed and immersion stressed (IMR) fish. Cross-section of climbing perch trunk kidney immunostained with NKAα1-antibody (green) showed region-specific immunostaining of renal ionocytes that include renal proximal ionocyte, renal distal ionocyte and renal collecting duct ionocyte. Immunofluorescence staining for NKAα1 is restricted to the basal membrane of renal epithelial cells in the proximal tubules that forms renal proximal ionocyte, whereas it extends to the basolateral membrane of renal epithelial cells in the distal and collecting tubules, which form renal distal and collecting duct ionocytes. Nuclei are counterstained with DAPI (blue). DIC and immunofluorescence images of NKA (green) and DAPI (blue) and its merged images were shown in each panel. Negative control kept under the same conditions without primary antibody that yielded no immunoreactivity was shown in the last panel. Scale bar = 10 μm.

**FIGURE 12:**
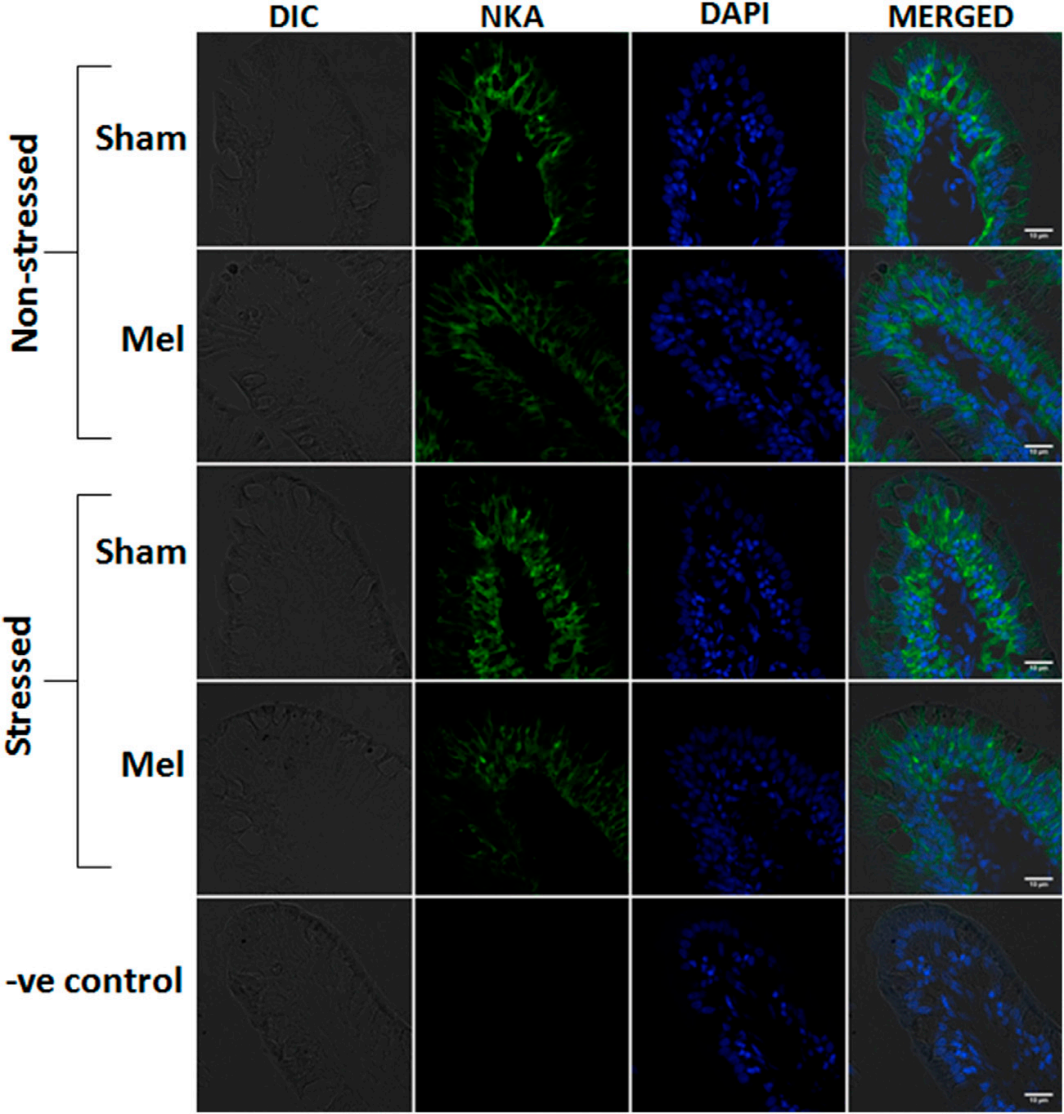
Confocal images showing the distribution of NKAα1-rich immunoreactivity in the intestinal villi of climbing perch after melatonin treatment (Mel) in nonstressed and immersion stressed (IMR) condition. NKA-immunostaining was seen on the basolateral sides of columnar epithelial cells, which act as entric ionocytes, and nuclei are counterstained with DAPI. Each panel consists of representative images of intestinal villi of fish from each group and starts with DIC image, fluorescence images of NKAα1 (green) and DAPI (blue) and its merged form in DIC. Last panel represents the corresponding images of negative control in which primary antibody incubation was omitted. Scale bar = 10 μm.

### 3.9 Integrative parabola model for melatonin-induced recovery response in hypoxia-stressed fish

**FIGURE 13:**
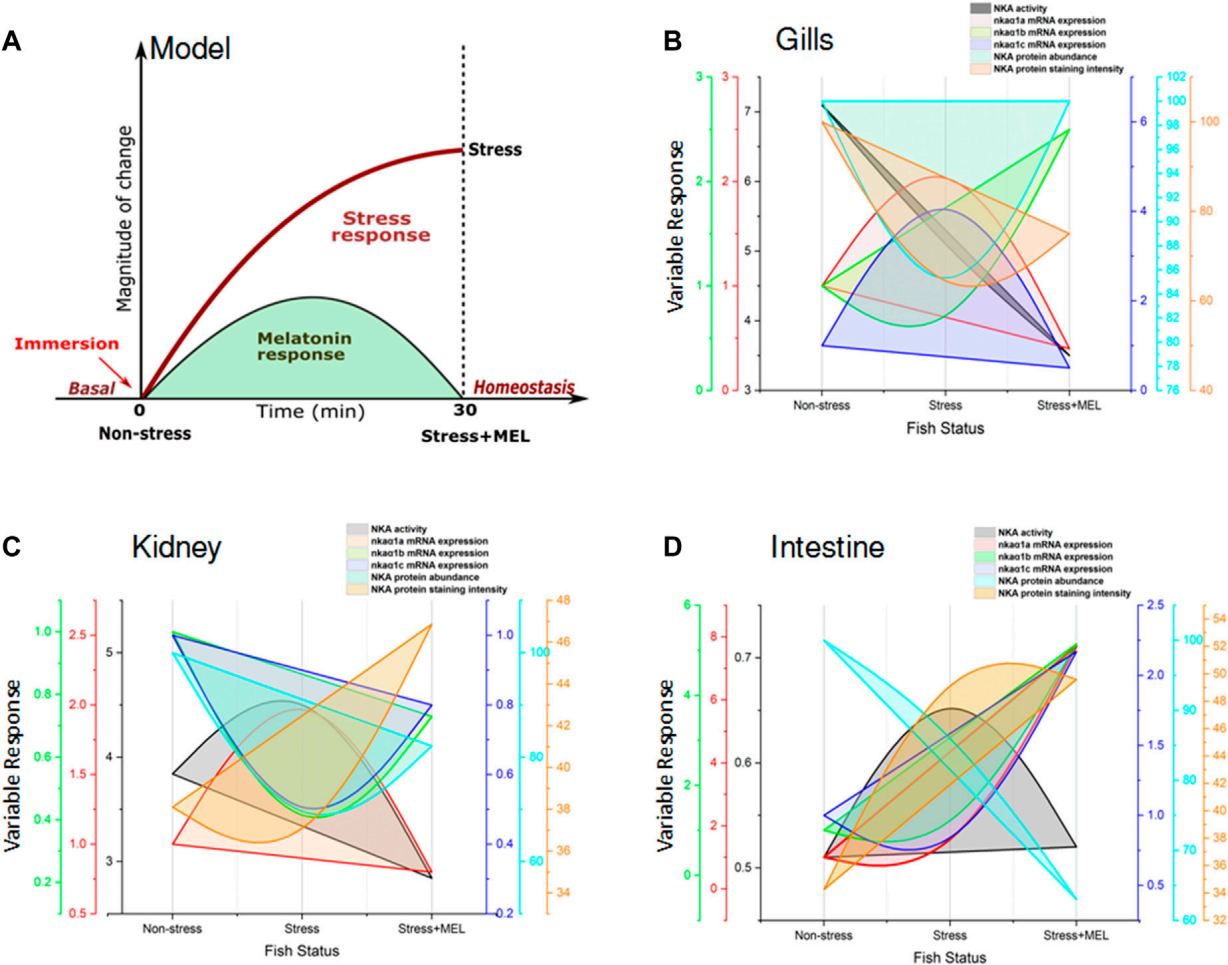
Model showing the NKA-driven recovery response by exogenous melatonin (Mel) in gills, kidney and intestine of immersion-stressed (IMR) climbing perch. The proposed model **(A)** represents a typical pattern of stress response in fish held for 30 min immersion stress against non-stressed condition. Immersion-stressed fish groups that received exogenous melatonin showed melatonin response as recovery response. In the subsequent figures **(B,D)**, the responses of tested NKA-regulatory parameters were plotted against three fish groups such as non-stressed, immersion-stressed and immersion stress plus melatonin. The response of gills **(B)**, kidney **(C)** and intestine **(D)** of these fish for time frame (0–30 min) were plotted on X-axis. The magnitude of changes in the NKA functions, quantified as NKA activity, isoform *nkaα1a*, *nkaα1b* and *nkaα1c* mRNA expression, NKAα protein abundance and NKA immunofluorescence staining intensity, were plotted using multi Y-line graphs in Origin 2018 software (Origin Lab, United States). Varied patterns of parabola that include complete, obliterate or weak were obtained for each tested parameter in gills, kidney and intestine based on its response to melatonin treatment in immersion-induced fish where melatonin tried to bring back the magnitude of tested parameters of stress-affected NKA regulatory function to basel level. Subsequent, comparative analysis of plotting patterns made for identifying the lead organ that integrate maximum recovery response to immersion stress was measured. A maximum score of 5/6 was obtained for kidney, whereas gills obtained a score of 4/6 and intestine secured 3/6. That depicted a lead role for kidney in the NKA-driven integration of Na^+^ homeostasis during melatonin intervention in immersion-stressed fish.

A hypothetical model was represented in Figure 13A showing a typical stress response in a hypoxic fish and how melatonin could induce recovery response in these fish. Subsequently, an integrative parabola model was constructed to generate the pattern of recovery response of test variables in non-stressed, immersion-stressed and immersion-stressed fish that received melatonin. Analysis of data on NKA specific activity, transcript expression of *nkaa1a, nkaa1b* and *nkaa1c,* NKA*a* protein abundance and immunofluorescence intensity of the test variables were done and that yielded formation of specific parabola for each variable tested in tissues such as gills (Figure 13B), kidney (Figure 13C) and intestine (Figure 13D). Qualitative analysis of the patterns of generated parabola showed complete, obliterate or weak type, based on its response to melatonin in bringing back the stress-disturbed NKA function to basal level. Comparative evaluation of these qualitative indices revealed total scores of 4/6, 5/6 and 3/6 for gills, kidney and intestine respectively (Table 4). Kidney epithelia secured maximum score on tested variables and this led us to conclude that kidney has a major lead role in melatonin-driven recovery response in this fish.

**TABLE 4:** Qualitative measurement of parabola of different tested variables generated from integrative parabola model based on the action of melatonin treatment in immersion-stressed climbing perch.

Tested variablesTissues	1	2	3	4	5	6	Scores obtained over total score of 6
Gills	0	1	0.5	1	1	0.5	4
Kidney	0.5	1	1	1	1	0.5	5
Intestine	1	0.5	0.5	0.5	0	0.5	3

Variable 1- NKA activity; 2- *nkaα1a* mRNA expression; 3- *nkaα1b* mRNA expression; 4- *nkaα1c* mRNA expression; 5- NKA protein abundance; 6- NKA protein staining intensity.

Type and score of parabola: Complete parabola- 1; Obliterate parabola- 0.5; Weak parabola- 0.

## 4 Discussion

In teleosts, the maintenance of systemic ionosmotic integration and subsequent ionic balance is mainly achieved by highly efficient ion transporter mechanisms that integrate the ion transport activities of gills, kidney and intestine (Evans et al., 2005; Peter, 2011; Peter and Gayathry, 2021). The paucity of information on the role of melatonin in the regulation of Na^+^ homeostasis in fish particularly during hypoxia stress, prompted us to investigate its short-term *in vivo* action on NKA functions in the ionocytes of air-breathing fish kept under water immersion. Here, we found a higher production of melatonin in hypoxic fish and a multidimensional regulation of NKA function in the tested ionocytes. This would help the hypoxic fish to recover or tolerate imbalance of systemic Na^+^ homeostasis and suggests a role for melatonin in stress and ease response in this fish.

The pineal melatoninergic system has shown a higher sensitivity to stress in vertebrates (Pfeffer et al., 2022). The substantial rise of melatonin in the plasma of hypoxic fish clearly indicated that a potential involvement of melatonin during stress response. Several studies have showed that melatonin might play a role in alleviating stress effects in teleosts, and in many cases it is related to the modulation of neuroendocrine responses within the HPI axis (Sánchez-Vázquez et al., 2019). Melatonin has been shown to modulate acute stress effects on zebrafish by inducing a sleep state, inhibiting cortisol levels, reducing locomotor activities, reducing lipid peroxidation and stimulating antioxidant enzymatic activity (Hacışevki and Baba, 2018; Lunkes et al., 2021). Treatments with melatonin at doses mimicking nocturnal increase of the hormonal levels are able to reduce stress effects in fish (Gesto et al., 2016). Likewsie, peripheral melatonin has been shown to inhibit the stress response in goldfish (*Carassius auratus*) and displays additional sedative effects in teleost (Azpeleta et al., 2010). Melatonin can exert its effects by acting through specific cellular receptors on the plasma membrane, similar to other hormones, or through receptor-independent mechanisms that involve complex molecular cross talk. The differential response of *mtnr1a*, *mtnr1bb* and its variant in the tested osmoregulatory epithelia of hypoxia-stressed fish and its response to exogenous melatonin further confirm the active involvement of melatoninergic system to hypoxia and its recovery response in this fish.

When kept for water immersion, the fish lost both [Na^+^] and [K^+^] contents and this would account for a typical stress response in these freshwater fish with the ability to breathe air. Interestingly, we found that melatonin treatment induced the recovery of these ions loss in hypoxia-stressed fish. The restoration of [Na^+^] content in hypoxic fish by melatonin could be due to its integrative action on NKA functions in the primary ionocytes located in the osmoregulatroy tissues. This evidence along with the rise in melatonin level observed in the plasma of hypoxic fish indicates a role for melatonin in both stress response and recovery or ease response in this fish. The drop in [K^+^] content in non-stressed fish after melatonin treatment and its recovery in hypoxic fish by melatonin clearly indicate the sensitivity of K^+^ ion to melatonin and its protective action against hypoxic tolerance in fish. It is apparent that freshwater fish loose ions during their encounter with stressors (Wendelaar Bonga, 1997; Evans et al., 2005; Peter, 2011). Melatonin also drives a lower availability of extracellular K^+^ in our fish but enhances the availability of more free or net Ca^2+^ in non-stressed fish, emphasizing its role in ion transport. But the higher demand for free Ca^2+^ is retained in fish that received both melatonin and immersion stress and that point to the ability of melatonin to ensure tight cellular Ca^2+^ signaling in both stressed and non-stressed conditions.

### Melatonin action on branchial ionocytes

The activity pattern of NKA has been extensively used as a measure of hydromineral capacity in teleosts (Dang et al., 2000; McCormick, 2001; Peter et al., 2011). In fishes, many endocrine signals including thyroid hormones and cortisol have been shown to contribute systemic hydromineral balance by modulating NKA transporter functions during adverse stress conditions (Dang et al., 2000; Peter, 2011; Peter and Peter, 2011). As the most important ionoregulatory organ in fish, gills serves as a sensitive target of stress in teleosts (Evans et al., 2005; Peter and Peter, 2011). The rise in NKA activity in the gills after melatonin treatment in non-stressed fish points to the direct action of melatonin on NKA function in gills and its ability to modify brachial Na^+^ transport. A rapid modulation of NKA activity in response to various environmental challenges has been reported in osmoregulatory tissues of fish species (Mancera and McCormick, 2000), including climbing perch (Simi et al., 2017).

Analysis of the functional *nkaα1* subunit isoforms such as *nkaα1a, nkaα1b* and *nkaα1c* in the gill epithelia showed a differential sensitivity to melatonin and hypoxia stress. The upregulation of mRNA expression of *nkaα1a* and *nkaα1c and* the downregulation of *nkaα1b* in branchial epithelia of non-stressed fish following melatonin treatment points to the ability of melatonin to differentially regulate *nkaα1* isoforms. Interestingly, these isoforms showed a reversed response to exogenous melatonin in immersion-stressed fish, providing transcriptomic evidence for a preferential action of melatonin on *nkaα1* isoform switching in stressed gill epithelia. Moreover, this differential isoform expression further confirms the direct genomic action of melatonin on selectively recruiting α1 isoforms to NKA function for recovering its altered NKA response to immersion-stress. In addition, the higher NKA*α* protein abundance in the gill epithelia by melatonin in both non-stressed and immersion-stressed fish reveals a translational regulation of NKA. It is likely that melatonin could rapidly activate both transcriptional and translational efficiency of NKA as it recruits more functional NKA*α-*protein for reestablishing Na^+^ transport in gill epithelia of hypoxic fish.

NKA phosphorylation as post-translational regulation is realized mainly *via* PKA, PKC or PKG (Vasilets et al., 1990; Fotis et al., 1999; Therien and Blostein, 2000; Poulsen et al., 2012). These kinases that depend on the type of α isoform and calcium concentration either inhibit or stimulate the pump activity (Cheng et al., 1999; Lopina, 2001). The activation of PKC-dependent NKA phosphorylation in gill epithelia of non-stressed and melatonin-treated fish points to the fact that melatonin could utilize the PKC switch to regulate NKA activity that shows an enhanced activity after melatonin treatment. Modulation of NKA activity due to the stimulation of protein kinases has also been demonstrated in fish tissues (Crombie, 1996; Tipsmark and Madsen, 2001). Interestingly, such modulation could be found in the stressed gills where melatonin favors PKA and PKG-dependent phosphorylation. It also appears that melatonin could utilize these kinase-switches to lower NKA activity in the gills of stressed-fish. Though there are reports on the protein kinases that have opposite effects on NKA activity (Therien and Blostein, 2000) or on NKA phosphorylation/dephosphorylation status due to the modifying protein kinases and protein phosphatases activities (Ewart and Klip, 1995; Therien and Blostein, 2000) (Helenius et al., 2010).

Branchial ionocytes are localized in fish gill epithelia using NKAα-subunit antibody (Wilson and Laurent, 2002; McCormick et al., 2009). The prominent NKAα-immunoreactivity in gill epithelia that marks the presence of branchial ionocytes by both immunofluorescence and immunohistochemical assays indicate that these cells are located on the surface of secondary gill lamellae of climbing perch as demonstrated earlier (Peter et al., 2011). The enhanced branchial NKAα-immunoreactivity after melatonin in non-stressed and hypoxia-stressed fish further indicates a targeted action of melatonin on branchial ionocyte function in these fish. In addition, it is likely that the dispersed NKAα-immunoreactivity in the branchial ionocytes of hypoxia-stressed fish may appear to be due to an increased gill permeability associated with an increased catecholamine secretion as similar catecholamine-induced rise in gill permeability has been found in hypoxia-stressed rainbow trout (McNeill and Perry, 2006). It seems that the higher NKAα-immunoreactivity that reveals elevated protein abundance in these cells of immersion-stressed fish by melatonin could be due to the protective action of melatonin in rescuing ionocyte functions during immersion stress. It also appears that melatonin could help these branchial ionocytes to recruit more NKA*α* protein into their plasma membrane, favoring them to work against immersion-stress. This view agrees with the earlier studies on the protective action of melatonin as it decreases the level of plasma catacholamines in birds (Mahata and De, 1991), offers protective action against adrenaline-induced stress injury in rats (Rudra et al., 2014). Similarly, melatonin administration seems to protect the mice from restraint-stress (SajeebKhan and Peter, 2015). In addition, exogenous melatonin has been shown to protect adrenal glands in rats (Pertsov, 2006) and is essential for homeostatic control of energy metabolism in several vertebrate groups including fish (Conde-Sieira et al., 2012).

### Melatonin action on renal epithelia

Kidney plays a pivotal role in maintaining ionic regulation in fishes (Varsamos et al., 2005; Peter, 2011). But its role in the transport of Na^+^ is less studied in freshwater fishes particularly during stress conditions. It has been shown that hypoxia induction leads to modified pattern of NKA function in kidney of fish (Simi et al., 2017). It appears that melatonin has a direct control on renal sodium transport as it favored the decline in the NKA activity in kidney tubules of immersion-stressed fish though it stimulated its activity in renal epithelia of non-stressed fish. This view further supports the notion that melatonin could favor hyperosmoregulatory ability of this fish as evident in its ability to enhance the arterial blood [Na^+^] levels. It is well known that fishes regulate their water and mineral balance by promoting hyperosmoregulatory mechanisms in freshwater (Evans et al., 2005). The analysis of mRNA expression of *nkaa1a, 1b* and *1c* genes in renal epithelia of non-stressed fish that showed differential regulation after melatonin treatment indicate a preferential action of melatonin. Moreover, similar to gills, melatonin produced a rise in *nkaa1a* isoform expression in the kidney of non-stressed fish which later showed regulation in hypoxic fish. Likewise, *nkaa1b* and *nkaa1c* that showed upregulation in stressed kidney in response to melatonin further confirms the ability of melatonin to regulate the NKA-driven Na^+^ transport in the kidney tubules by differentially regulating its *nkaα1* isoform diversity.

The rise in NKA*α-*protein expression along with an intensified NKAα-immunoreactivity in proximal and distal renal ionocytes, further support a direct action of melatonin on NKAα-protein in kidney epithelia during hypoxia condition. This would enable the renal tubules to recover the disturbed systemic Na^+^ homeostasis back to basal level in hypoxia-induced fish. This further reveals a direct translational control of melatonin on NKAα-subunit function and also points to the ability of melatonin to promote the reabsorption of Na^+^ in the renal epithelia during recovery response. This view is consistent with the reports that melatonin can increase both water and Na^+^ filtration rates in Wister rats (Pishak and Kokoshchuk, 1995), though a diminished epithelial Na^+^ transport has been reported after melatonin treatment in baboons (Legris et al., 1982).

Phosphorylation of different positions of NKAα1 subunit determines inhibition or activation of NKA activity in renal epithelia (Pedemonte et al., 1997; Ogimoto et al., 2000). It appears that melatonin could regulate NKA pump activity by way of inactivating PKC-dependent phosphorylation in non-stressed renal epithelia. The lowered NKA activity by melatonin in this tissue of stressed fish without altering its phosphorylation efficiency also points to a multi-targeted action of melatonin in kidney epithelia. This would further points the abilty of melatonin to modify epithelial Na^+^ and K^+^ transport functions during hypoxia conditions which could also demand a lesser sensitive kinases-activated phosphorylation. Studies have shown that PKA-mediated phosphorylation at Ser^938^ regulates NKA activity in rat proximal tubules after rapid activation of angiotensin-II, which is a stimulant of Na^+^ reabsorption in proximal and distal tubules (Massey et al., 2016). Likewise, involvement of dopamine and angiotensin-II in stimulating NKA function has been shown mediated by PKC at Ser^18^ (Zhang et al., 2010).

The higher NKA*α*-immunoreactivity in renal proximal and distal ionocytes after melatonin treatment indicates that melatonin enhances NKA*α* protein abundance in the renal tubules. Immunohistochemical studies demonstrated that NKA*α* is localized mainly in the epithelia of kidney tubules as in many teleosts (Ura et al., 1996; Lin et al., 2004). The NKA*α*-immunoreactivity in different segments of nephron except glomerulus indicates that these tubules are responsible for the reabsorption of salts. Distinct subtypes of renal ionocytes namely; proximal, distal and collecting tubular ionocytes have been found in this fish that showed their specific structural peculiarities (Peter and Gayathry, 2021). The diversified NKA*α*-immunoreactivity pattern among the ionocytes in renal tubules of non-stressed fish after melatonin may correspond to the functional attributes of ionocytes.

### Melatonin action on enteric epithelia

In freshwater teleosts, as part of the integrative control of systemic ion homeostasis, intestinal epithelia can compensate the potential loss of Na^+^ ion across branchial and renal epithelia *via* accelerating Na^+^ ion uptake (Peter, 2011). It appears that melatonin, despite its abundant availability in intestinal tissue as one of the important extra-pineal sources of melatonin synthesis (Bubenik, 2002; Mukherjee and Maitra, 2015), had little action on the total NKA activity of this tissue. But it is interesting that melatonin could induce a differential action on *nkaα1* isoforms in intestinal tissue that showed upregulation in hypoxic fish, while showing downregulation in non-stressed fish. In addition, a probable transcriptomic activation of *nka*α1 isoforms could be seen due to the lowered protein abundance and a PKC–mediated NKA phosphorylation. The lowered NKA*α*-immunoreactivity and immunoblotting in intestinal epithelia that corresponds to unaffected NKA*α* protein abundance during hypoxia reflect a lower participation of intestinal NKA function in these fish probably as part of integrative control of ion homeostasis driven by melatonin. However, it is likely that melatonin can involve in hypoxic tolerance by way of modulating the transcriptomic and post-translational modifications of NKA functions. It also appears that melatonin could rely more on kidney than gills and intestine to recover the disturbed systemic Na^+^ homeostasis in the face of immersion stress. The earlier reports on the presence of melatonin receptors in fish intestinal villi support our observation that melatonin can modify the transmembrane transport of Na^+^ transport. Further, melatonin has been shown to induce relaxation of gut muscles to facilitate intestinal motility in rats (Bubenik, 2008). Similarly, it interacts with dopamine-sensitive Ca^2+^-activated K^+^channels in intestine (Reyes-Vázquez et al., 1997). Likewise a protective role of melatonin against stress-induced gastric lesions in rats (Cho et al., 1989) and the restoration of microcirculation (Konturek et al., 2011) have been presented. Exogenous melatonin has been shown to reduce diarrhea in rats with colitis (Cuzzocrea et al., 2001) and known to play a role in regulating Cl^−^ secretion in the colon (Chan et al., 1998).

### Integration and recovery of ionocyte function by melatonin in hypoxia-stressed fish

It appears that melatonin integrates multidimensional regulation of NKA functions in fish ionocytes that operates at transcriptional, translational and post-transcriptional levels. This evidence for a role of melatonin in the integration of systemic Na^+^ homeostasis demands activation/deactivation of NKA functions and subsequently the rate of Na^+^/K^+^ transport across the ionocytes to establish an optimal whole-body ionosmotic status during stress and ease conditions.

As major integral membrane transporter that establishes cellular and systemic homeostasis, NKA drives Na^+^ signaling in the osmoregulatory ionocytes. The integrative action of melatonin on NKA-driven Na^+^ homeostasis was tested in gill, kidney and intestinal epithelia by quatifying the ouabain-sensitive NKA activity, its transcriptional expression, NKA *α-*immunoreactivity and protein density and the phosphorylation status. We found that melatonin exerts an integrative action on primary ionocytes such as branchial, renal and enteric ionocytes that showed spatial and differential responses to immersion stress. Melatonin regulates the stress-induced alterations of NKA activity, *nkaα1a, 1b* and *1c* isoforms expression, NKA *α-*immunoreactivity and NKA *α*-protein expression in the tested epithelia, indicating a rapid and direct control of melatonin on NKA-transcriptional and translational regulations. Furthermore, melatonin produced differential and spatial regulation of NKA by evoking phosphorylation of protein kinases in both non-stressed and stress-challenged fish, indicating a tissue-specific post-translational regulation of NKA function that demands PKC, PKA or PKG signaling pathways. This is consistent with the report that hormones can create acute changes in NKA pump activity *via* either by regulating subcellular distribution of NKA pump-units or exerting reversible phosphorylation of the catalytic subunit (Ewart and Klip, 1995).

The generated integrative parabola model demonstrated that a pattern of recovery response of melatonin occurs in hypoxia stressed fish. This model provides quantitative evidence for the action of melatonin that recover the stress-induced disturbance in NKA function based on the response pattern of test variables in hypoxia-stressed fish that received melatonin. The analysis of the patterns of recovery response that occurred during NKA regulation resulted in varied types of parabola patterns for the tested variables in the tissues. Kidney epithelia that scored maximum points on tested variables, led us to conclude that kidney, followed by gills and intestine, plays a major lead role in melatonin-driven recovery response in this fish. It is evident that kidney could utilize its major NKA regulatory mechanisms in response to melatonin-driven recovery action in stressed fish compared to its partners such as gills and intestine. Overall, the study provides evidence that melatonin 1) targets and directs Na^+^ homeostasis by differentially regulating NKA functions in ionocytes and 2) integrates the NKA-driven ionocyte functions of gills, kidney and intestine with a lead role for kidney to recover the disturbed NKA functions during hypoxia stress. Our data thus support the earlier hypothesis (Peter, 2013) that melatonin can promote ease response in fish during their acclimation to stress, thus considering melatonin as a recovery or ease hormone in fish.

## Data Availability

The raw data supporting the conclusion of this article will be made available by the authors, without undue reservation.
